# Modulation of Autoimmune T-Cell Memory by Stem Cell Educator Therapy: Phase 1/2 Clinical Trial

**DOI:** 10.1016/j.ebiom.2015.11.003

**Published:** 2015-11-05

**Authors:** Elias Delgado, Marcos Perez-Basterrechea, Beatriz Suarez-Alvarez, Huimin Zhou, Eva Martinez Revuelta, Jose Maria Garcia-Gala, Silvia Perez, Maria Alvarez-Viejo, Edelmiro Menendez, Carlos Lopez-Larrea, Ruifeng Tang, Zhenlong Zhu, Wei Hu, Thomas Moss, Edward Guindi, Jesus Otero, Yong Zhao

**Affiliations:** a Endocrinology Section, Department of Medicine, University de Oviedo, Oviedo 33006, Spain; b Endocrinology and Nutrition Service, Hospital Universitario Central de Asturias, Oviedo 33011, Spain; c Unit of Transplants, Cell Therapy and Regenerative Medicine, Hospital Universitario Central de Asturias, Oviedo 33011, Spain; d Cellular Biology of Renal Diseases Laboratory, Instituto de Investigación Sanitaria Fundación Jiménez Díaz, Universidad Autónoma de Madrid, Madrid 28049, Spain; e Section of Endocrinology, The First Hospital of Hebei Medical University, Shijiazhuang 050031, PR China; f Hematology and Hemotherapy Service, Hospital Universitario Central de Asturias, Oviedo 33011, Spain; g Department of Immunology, Hospital Universitario Central de Asturias, Oviedo 33011, Spain; h Fundación Renal “Iñigo Álvarez de Toledo”, Madrid 28003, Spain; i Department of Hepatobiliary Surgery, The Fourth Hospital of Hebei Medical University, Shijiazhuang, Hebei 050031, PR China; j Department of Pathology, The First Hospital of Hebei Medical University, Shijiazhuang, Hebei 050031, PR China; k Department of Research, Hackensack University Medical Center, Hackensack, NJ 07601, USA; l CORD:USE Cord Blood Bank, Orlando, FL 32810, USA

**Keywords:** AIRE, autoimmune regulator, CB-SCs, human cord blood-derived multipotent stem cells, CCR7, C–C chemokine receptor 7, HbA_1_C, glycated hemoglobin, HLA, human leukocyte antigen, IL, interleukin, MLR, mixed leukocyte reactions, MNC, mononuclear cells, M2, muscarinic acetylcholine receptor 2, OGTT, oral glucose tolerance test, PBMC, peripheral blood mononuclear cells, R, responder, SCE, Stem Cell Educator, S, stimulator, T_CM_, central memory T cells, TCR, T-cell receptor, T_EM_, effector memory T cells, TGF-β1, transforming growth factor-β1, Th, helper T cell, T1D, type 1 diabetes, Tregs, regulatory T cells, Type 1 diabetes, Autoimmunity, Memory T cells, Cord blood stem cell, Immune modulation

## Abstract

**Background:**

Type 1 diabetes (T1D) is a T cell-mediated autoimmune disease that causes a deficit of pancreatic islet β cells. The complexities of overcoming autoimmunity in T1D have contributed to the challenges the research community faces when devising successful treatments with conventional immune therapies. Overcoming autoimmune T cell memory represents one of the key hurdles.

**Methods:**

In this open-label, phase 1/phase 2 study, Caucasian T1D patients (N = 15) received two treatments with the Stem Cell Educator (SCE) therapy, an approach that uses human multipotent cord blood-derived multipotent stem cells (CB-SCs). SCE therapy involves a closed-loop system that briefly treats the patient's lymphocytes with CB-SCs in vitro and returns the “educated” lymphocytes (but not the CB-SCs) into the patient's blood circulation. This study is registered with ClinicalTrials.gov, NCT01350219.

**Findings:**

Clinical data demonstrated that SCE therapy was well tolerated in all subjects. The percentage of naïve CD4^+^ T cells was significantly increased at 26 weeks and maintained through the final follow-up at 56 weeks. The percentage of CD4^+^ central memory T cells (T_CM_) was markedly and constantly increased at 18 weeks. Both CD4^+^ effector memory T cells (T_EM_) and CD8^+^ T_EM_ cells were considerably decreased at 18 weeks and 26 weeks respectively. Additional clinical data demonstrated the modulation of C–C chemokine receptor 7 (CCR7) expressions on naïve T, T_CM_, and T_EM_ cells. Following two treatments with SCE therapy, islet β-cell function was improved and maintained in individuals with residual β-cell function, but not in those without residual β-cell function.

**Interpretation:**

Current clinical data demonstrated the safety and efficacy of SCE therapy in immune modulation. SCE therapy provides lasting reversal of autoimmune memory that could improve islet β-cell function in Caucasian subjects.

**Funding:**

Obra Social “La Caixa”, Instituto de Salud Carlos III, Red de Investigación Renal, European Union FEDER Funds, Principado de Asturias, FICYT, and Hackensack University Medical Center Foundation.

## Highlights

• We prove the safety and feasibility of a two-treatment approach with SCE therapy.
• The percentage of naïve CD4^+^ and T_CM_ cells were increased, but the percentage of CD4^+^ T_EM_ and CD8^+^ T_EM_ cells were declined.
• The expression of CCR7 on naïve T and T_CM_ cells were markedly increased.

The current studies demonstrated the safety and feasibility of a two-treatment approach with Stem Cell Educator therapy and well tolerated in all subjects, without significantly changing the numbers and ratios of different cell compartments in the subjects' immune system. Both the percentage of CD4^+^ and CD8^+^ effector memory T cells were substantially decreased in the peripheral blood of T1D subjects, whereas the percentage of CD4^+^ central memory T cells was markedly and constantly increased. These findings provide a solution to alter the autoimmune memory compartment in T1D, while mitigating safety and ethical concerns associated with conventional immune therapies.

## 1 Introduction

Type 1 diabetes (T1D) is a major global health issue, and its incidence is increasing. T1D is a T cell-mediated autoimmune disease that reduces the population of pancreatic islet β cells, which limits insulin production and interferes with glucose homeostasis. The immune dysfunction in T1D is complicated, with effects both in pancreatic islets and outside the pancreas. Different components of the immune system [e.g., CD4^+^, CD8^+^ T cells, Tregs, B cells, dendritic cells (DCs), monocyte/macrophages (Mo/Mϕs), natural killer T cells (NKTs)] contribute to autoimmune responses in T1D, complicating efforts to develop successful treatments or a cure that will work across most or all individuals with the disease. Several recent clinical trials (Bach, 2011, Wherrett et al., 2011) highlight the challenges in conquering T1D, but their failures provide some valuable lessons about the limitations of conventional immune therapy and the future direction of the quest. Specifically, they point to the need for an approach that produces comprehensive immune modulation at both the local pancreatic and systematic levels rather than targeting the pancreatic effects of one or a few components of the immune system. The Stem Cell Educator therapy takes this broader approach (Zhao and Mazzone, 2010, Zhao et al., 2012, Zhao, 2012, Zhao et al., 2013, Li et al., 2015).

Physiologically, the human immune system constantly protects the body against a variety of pathogens that may be encountered. Following the recognition and eradication of pathogens through adaptive immune responses, the majority (90–95%) of T cells undergo apoptosis with the remaining cells forming a pool of memory T cells, designated central memory T cells (T_CM_), effector memory T cells (T_EM_), and resident memory T cells (T_RM_) (Clark, 2015). In comparison to conventional T cells, these memory T cells are long-lived with distinct phenotypes, such as expression of specific surface markers, rapid production of different cytokine profiles, capability of direct effector cell function, a different potential for proliferation, and unique homing distribution patterns. As a group, memory T cells display quick reactions upon re-exposure to their cognate antigens in order to eliminate the reinfection of pathogens and restore balance and harmony of the immune system. Nevertheless, increasing evidence establishes that autoimmune memory T cells become the “stumbling blocks” and hinder most attempts to treat or cure autoimmune diseases, including T1D, multiple sclerosis (MS), rheumatoid arthritis (RA), and system lupus erythematosus (SLE) (Ehlers and Rigby, 2015, Clark, 2015, Devarajan and Chen, 2013). Therefore, novel and more comprehensive approaches are needed to fundamentally correct the inordinate dominance of autoimmune T cell memory and overcome the complexities of autoimmune responses.

We previously characterized a new type of stem cell from human cord blood, designated a cord blood-derived multipotent stem cell (CB-SCs) (Zhao et al., 2006, Zhao and Mazzone, 2010). CB-SCs display a unique phenotype with both embryonic and hematopoietic markers that distinguish them from other known stem cell types, including hematopoietic stem cells (HSCs), mesenchymal stem cells (MSCs), and monocytes/macrophages (Mo/Mϕ) (Zhao et al., 2003). SCE therapy functions as an “artificial thymus” that circulates a patient's blood through a blood cell separator, briefly treats the patient's lymphocytes with CB-SCs in vitro, induces immune tolerance through the action of autoimmune regulator (AIRE, expressed by CB-SCs), returns the educated autologous lymphocytes to the patient's circulation, and restores immune balance and homeostasis (Zhao and Mazzone, 2010, Zhao et al., 2012, Zhao, 2012). This approach was piloted in clinical studies for the treatment of diabetes and other autoimmune diseases in China with patients of Chinese origin (Zhao et al., 2012, Zhao, 2012, Zhao et al., 2013, Li et al., 2015). Our clinical data demonstrates that the SCE therapy provides long-lasting reversal of autoimmunity that induces the regeneration of pancreatic islet β cells and improvement of metabolic control in individuals with longstanding T1D (Zhao et al., 2012). Findings from recent autoimmune-caused Alopecia Areata (AA) trial provide visible evidence that SCE therapy can control autoimmunity and lead to the regeneration of tissues like hair regrowth (Li et al., 2015). Here, we explored the expansion of the therapeutic potential of the SCE therapy to the treatment of Caucasian T1D subjects in Spain.

## 2 Methods

### 2.1 Patients

**Fig. 1:**
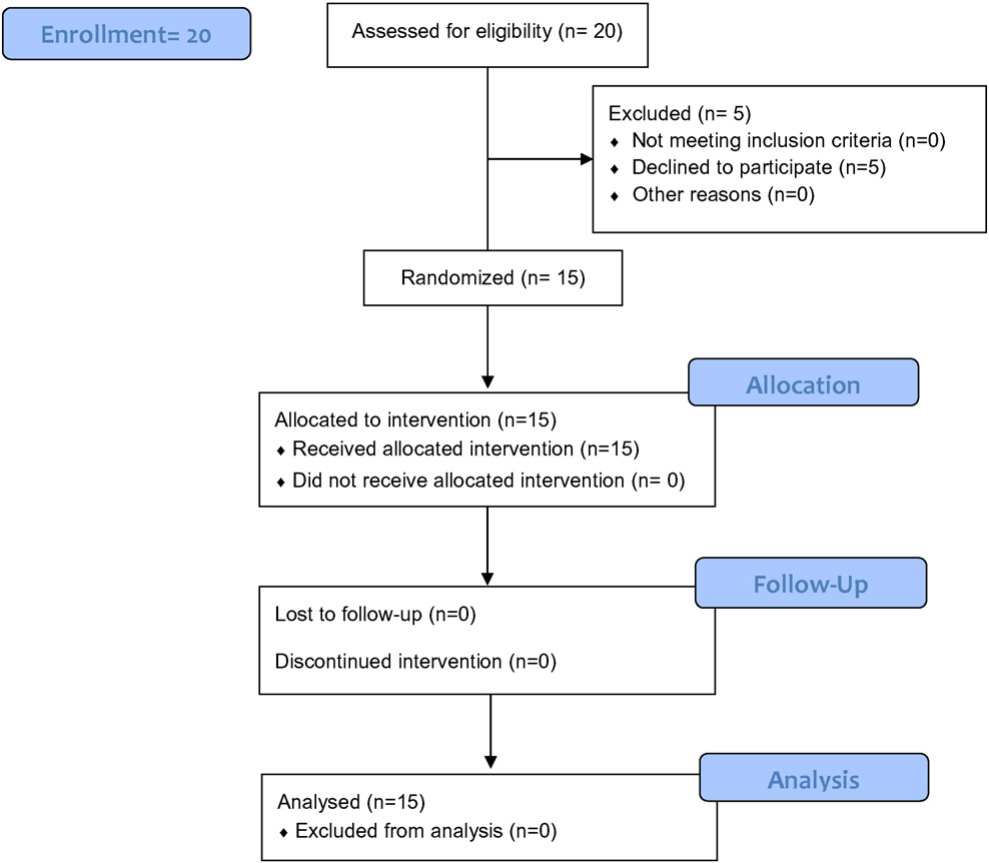
Study flow chart.

T1D patients receiving care at the Endocrinology and Nutrition Service, Hospital Universitario Central de Asturias (Oviedo, Spain) were enrolled in this phase 1/phase 2, open-label clinical trial conducted from November 27, 2012 through October 1, 2014. The principal investigator designed the clinical trial and received ethical approval for the clinical treatment protocol and consent form from Regional Committee for Clinical Research Ethics and the Comisión Permanente de Trasplantes del Consejo Interterritorial del Sistema Nacional de Salud. The signed informed consent was obtained from each participant. The clinical trial was conducted in 15 subjects with established T1D (Table 1). Subjects were qualified for recruitment if they met the 2012 diagnosis standards of the American Diabetes Association (ADA) and if a blood test indicated the presence of at least one autoantibody to pancreatic islet β cells (Fig. 1). Key exclusion criteria included clinically significant liver (AST or ALT 2 ≥ x upper limit of normal), kidney (creatinine ≥ 2.0 mg/dl), or heart disease; pregnancy or breastfeeding mothers; immunosuppressive medication; known active infection with viral diseases; or diseases associated with immunodeficiency; or hemoglobin < 10 g/dl or platelets < 100 k/ml; use of immunosuppressive medication within one month.

### 2.2 SCE Therapy and Follow-up

All the participants received two treatments with the SCE (Tianhe Stem Cell Biotechnology®, USA). The preparation of CB-SC cultures and SCEs was performed as previously described (Zhao et al., 2012). Briefly, human cord blood units derived from healthy allogeneic donors were obtained from Centro Comunitario de Sangre y Tejidos de Asturias (CCST, Oviedo, Spain). All cord blood samples were screened for HIV I&II, HBsAg, HBcAg, HCV, HIVNAT, STS, HBVNAT, HCVNAT, HTLV I/II, West Nile, Chagas, and CMV, and only pathogen-free cord blood units were used for clinical treatment. Human CB-SCs were produced as previously described (Zhao et al., 2006, Zhao et al., 2009) with the following modifications. Cord blood mononuclear cells were plated in SCE devices in serum-free culture medium (Lonza, Walkersville, MD) and incubated at 37 °C, in 8% CO_2_. After 2–3 weeks, CB-SCs growing at 90% confluence (about 10^7^ cells/device) were prepared for clinical trial. CB-SCs were characterized by flow cytometry, using markers such as the leukocyte common antigen CD45 and embryonic stem (ES) cell-specific transcription factor OCT3/4 (Fig. S1). The measured endotoxin level was < 0.05 EU/ml. One Educator device was generated from one cord blood unit, and used for one subject at one treatment.

**Fig. 2:**
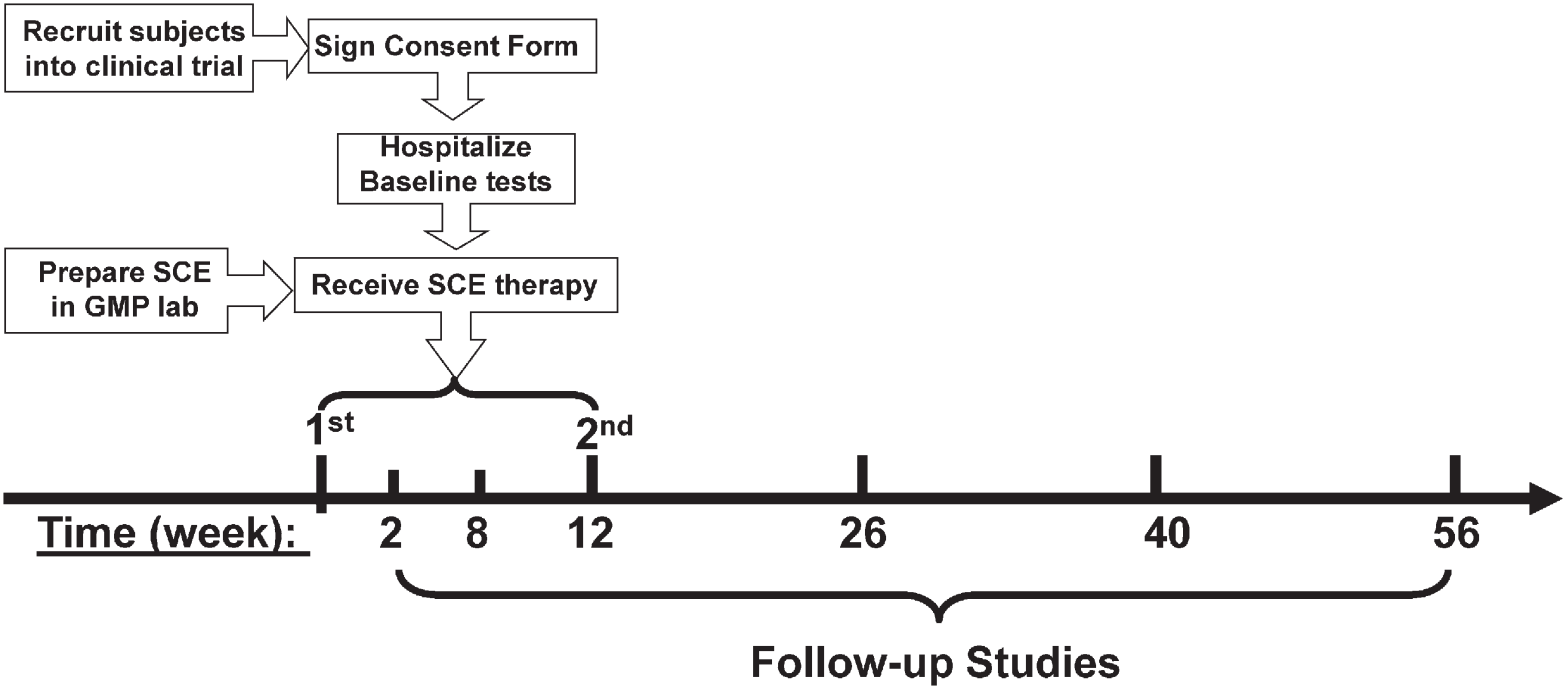
*Diagram of SCE therapy for the treatment and follow-up studies*. All the participants received two treatments with the SCE therapy. Human cord blood units were derived from healthy allogeneic donors. The preparation of CB-SC cultures SCE devices were cultured in serum-free culture medium and incubated at 37 °C, in 8% CO_2_. After 2–3 weeks, CB-SCs growing at 90% confluence were prepared for clinical trial. One Educator device was generated from one cord blood unit, and used for one subject at one treatment. Follow-up visits were scheduled 2, 8, 12, 18, 26, 40 and 56 weeks after treatment for clinical assessments and laboratory tests. Previous work demonstrated that participants receiving sham therapy failed to show changes in immune modulation (Zhao et al., 2012).

For the SCE therapy (Zhao et al., 2012, Zhao et al., 2013), a 16-gauge IV needle was placed in the left (or right) median cubital vein, and the patient's blood was passed through a Blood Cell Separator MCS + (Haemonetics®, Braintree, MA) to isolate mononuclear cells (MNC) in accordance with the manufacturer's recommended protocol. For a single session of MNC collection by apheresis, approximately 10 l of blood was processed from each enrolled subject within 6–7 h, with the collection of about 1 × 10^10^ MNCs. The isolated mononuclear cells were transferred into the SCE device for the treatment with allogeneic CB-SCs, and other blood components were automatically returned to the patient's circulation. In the SCE device, MNC ioslated from a patient's peripheral blood were slowly passed through the stacked discs with adherent CB-SCs at the CB-SCs:MNC ratios from 1:20 to 1:50. After interaction for 2–3 h (Fig. S2), CB-SC-treated mononuclear cells were returned to the patient's blood circulation via a dorsal vein in the hand with physiological saline. It took 8–9 h. Patients were hospitalized for one day to monitor temperature and conduct blood count tests for adverse reactions following treatment. After 3 months, all subjects received a 2nd treatment with SCE therapy, as described above. Follow-up visits were scheduled 2, 8, 12, 18, 26, 40 and 56 weeks after treatment for clinical assessments and laboratory tests (Fig. 2). Previous work demonstrated that patients receiving sham therapy did not show changes in immune modulation (Zhao et al., 2012).

To evaluate the β-cell function, fasting and glucagon-stimulated C-peptide levels were examined at baseline and after treatments with SCE therapy. The glucagon-stimulated C-peptide test was performed as previously described (Gjessing et al., 1987). Glucagon (1 mg, i.v.) was administrated within 30 s, and six minutes later, plasma samples were collected for the C-peptide test by Ultrasensitive C-peptide ELISA kit (Mercodia, Uppsala, Sweden).

### 2.3 Study End Points

The primary study end points were feasibility and safety of the SCE therapy through 56 weeks post-treatment and preliminary evaluation of the efficacy of the therapy for changing immune markers in T1D subjects. The secondary study end point was preliminary evidence for efficacy of the therapy in the improvement of β-cell function. Baseline blood samples were collected prior to SCE therapy.

### 2.4 Mixed Leukocyte Reactions (MLR) and Ex Vivo Co-cultures

Human buffy coat blood units were purchased from the Blood Center of New Jersey (East Orange, NJ). Human peripheral blood-derived mononuclear cells (PBMC) were harvested as previously described (Zhao et al., 2012, Zhao et al., 2013). To examine the immune modulating effects of CB-SCs on T cells via the mixed leukocyte reactions (MLR), responder cells were co-cultured with allogeneic stimulator cells irradiated at 3000 rad at the R:S ratio of 1:2, in the presence or absence of CB-SCs. The ratio of CB-SCs:responder was 1:10. After 4–5 days of co-culture, cells were photographed with an Olympus IX71 inverted microscope and collected for flow analysis.

To analyze the CCR7 expression on CD45RO^+^ CD62L^−^ T_EM_ cells, adult peripheral blood-mononuclear cells (PBMCs) were cocultured with CB-SCs at the CB-SCs:PBMCs ratio of 1:10 in serum-free culture medium (Lonza, Walkersville, MD) and incubated at 37 °C, in 8% CO_2_. The untreated PBMCs served as control. The CB-SC-treated PBMCs were collected for flow cytometry at 24 and 48 h respectively.

To perform ex vivo studies, human cord blood units were provided by Cord:Use Cord Blood Bank (Orlando, FL). Only pathogen-free cord blood units were used for isolating CB-SCs. Human cord blood-derived stem cells (CB-SCs) were generated as previously described with the following modifications (Zhao et al., 2012, Zhao et al., 2013). Cord blood mononuclear cells were plated in serum-free culture medium (Lonza, Walkersville, MD) and incubated at 37 °C, in 8% CO_2_. After 2–3 weeks, CB-SCs growing at 80–90% confluence were prepared for co-culture with allogeneic lymphocytes.

### 2.5 Flow Cytometry

In preparation for the clinical trial, peripheral blood samples (10 ml per subjects) were obtained from patients before the treatment and at 2, 8, 18, 26 and 56 weeks respectively post-treatment. Cells were incubated with mouse anti-human mAbs (BioLegend, San Diego, CA), including PerCP/Cy5.5-conjugated anti-CD3, PerCP/Cy5.5-conjugated anti-CD4, PE-conjugated anti-CD8, FITC-conjugated anti-CD45RA, PE-conjugated anti-CD45RO, PE-conjugated anti-CD56, APC-conjugated anti-CCR7. To test the percentage and absolute cell numbers of different subsets in peripheral blood, cells were immunostained with BD MultiTEST reagents CD3 FITC/CD8 PE/CD45 PerCP/CD4 APC and CD3 FITC/CD16 + CD56 PE/CD45 PerCP/CD19 APC (BD Biosciences, San Jose, CA) according to the manufacture's recommended protocols. Isotype-matched mouse anti-human IgG antibodies (Beckman Coulter) served as a negative control for all fluorescein-conjugated IgG mAb. After staining, cells were collected and analyzed using a BD FACScalibur™ Cytometer. The final data were analyzed using the CellQuest Pro Software (Becton Dickinson, MD).

For ex vivo studies, flow cytometric analyses were performed as previously described (Zhao et al., 2009). Cells were stained for 30 min at room temperature and then washed with PBS prior to flow analysis. We used several mouse anti-human monoclonal Abs (mAbs), including APC-AF 750-conjugated anti-CD4, APC-AF 750- or Krome Orange-conjugated anti-CD8, PE- or FITC-conjugated anti-CD45RA, FITC-conjugated anti-CD45RO, ECD-conjugated anti-CD62L, and PE-Cy7-conjugated anti-CCR7. Isotype-matched mouse anti-human IgG antibodies (Beckman Coulter) served as a negative control for all fluorescein-conjugated IgG mAb. After staining, cells were collected and analyzed using a Gallios Flow Cytometer (Beckman Coulter), equipped with 3 lasers (488 nm blue, 638 red, and 405 violet lasers) for the concurrent reading of up to 10 colors. The final data were analyzed using the Kaluza Flow Cytometry Analysis Software (Beckman Coulter).

### 2.6 Statistics

An intention-to treat approach was used, with 15 patients undergoing SCE therapy. All participants were included in safety analyses. The feasibility of the SCE therapy was assessed by analyzing the number of the patients unable to complete the therapy and the number of patients who were lost to follow-up prior to the 12-month visit. The primary efficacy end points were the change in immune markers between baseline and follow-ups. Statistical analyses of data were performed by the two-tailed paired Student's t-test to determine statistical significance between baseline and follow-ups. Values were given as mean ± SD (standard deviation).

## 3 Results

### 3.1 Safety Profile and Feasibility of Two Treatments With SCE Therapy in Caucasian T1D Subjects

In previous clinical trials, all subjects received one treatment with the SCE therapy (Zhao et al., 2012, Zhao et al., 2013, Li et al., 2015). Due to the likelihood that significant numbers of pathogenic autoimmune cells may have remained in lymph nodes and other tissues, failing to enter into the bloodstream during the procedure, and thus may have escaped the exposure to CB-SCs, we added a second treatment three months following the initial session in these T1D subjects (n = 15, Table 1). No patients experienced any significant adverse events during the course of the two treatments with SCE therapy or during 56-week follow-up. During the procedure, only mild discomfort at the site of venipuncture (the median cubital vein) and some soreness of the arm were noted for some participants. No fever or rejection was noted during follow-up studies.

### 3.2 Clinical Efficacy of SCE Therapy in the Modulation of Memory T Cell Compartment of T1D Subjects

**Fig. 3:**
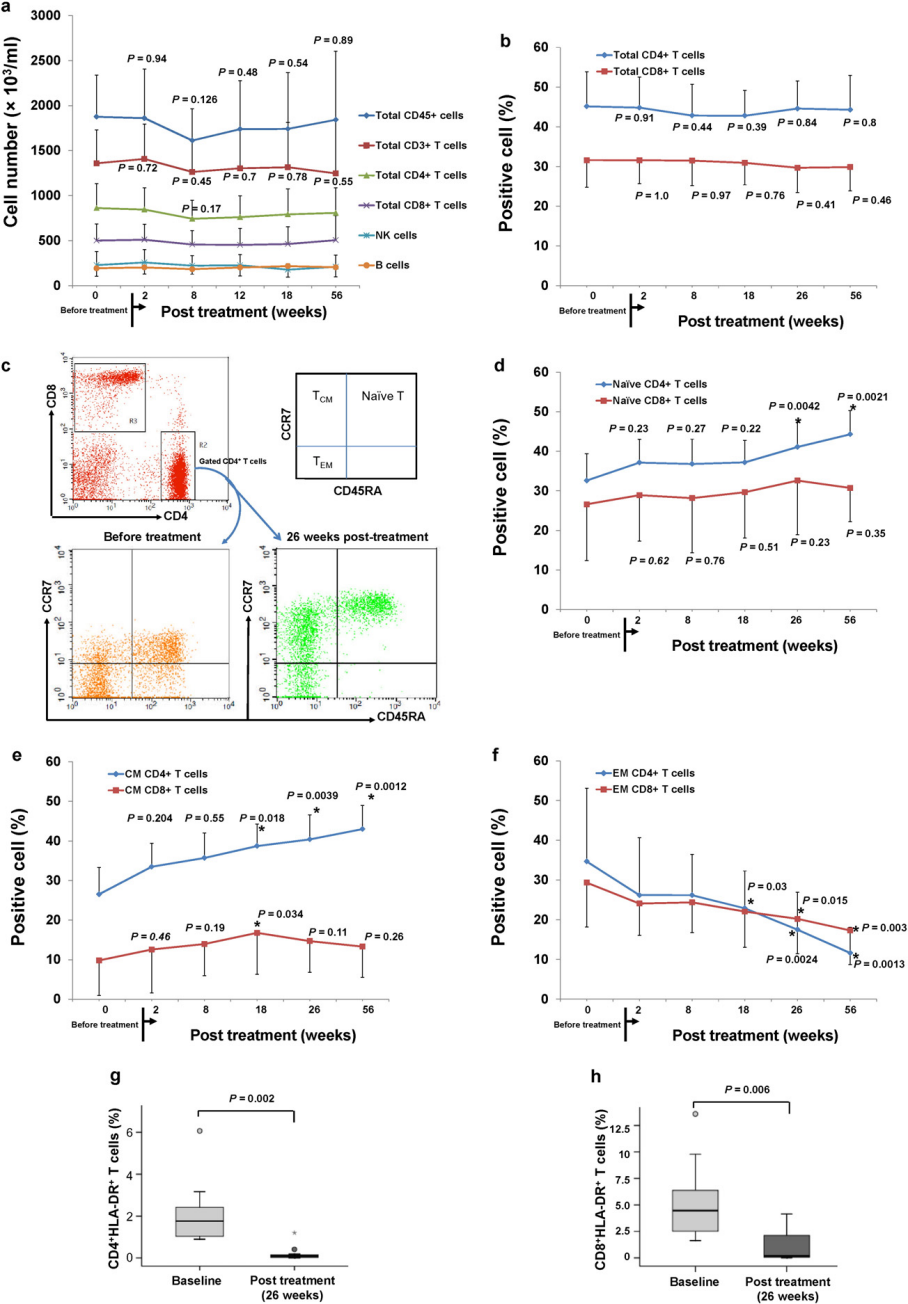
*Changes in immune markers in Caucasian T1D patients after SCE therapy*. All subjects received two treatments with SCE therapy. After 3 months, all subjects received a 2nd treatment with SCE therapy. Follow-up visits were scheduled 2, 8, 12, 18, 26, 40 and 56 weeks after treatment for clinical assessments and laboratory tests. Patient lymphocytes were isolated from peripheral blood by Ficoll-Hypaque (γ = 1.077) for flow cytometry analyses in T1D patients at baseline and different time points after SCE therapy. Isotype-matched IgG served as control. (*a*) Immune cell quantification in peripheral blood. (*b*) Percentage of CD4^+^ and CD8^+^ T cells in peripheral blood. (*c*) Outline of the markers and approach for the characterization of different T-cell subpopulations. CD45RA and CCR7 were applied to characterize the naïve and memory T cells in the gated CD4^+^ (R2) T cells. Flow cytometry showed the baseline levels of T-cell populations (bottom left panel, orange) and those at 26 weeks post-treatment (bottom right panel, green) in the PBMCs of T1D patient. (*d*) Flow Analysis of naïve CD4^+^ and CD8^+^ T cells in peripheral blood, demonstrating an increase in the percentage of naïve CD4^+^ T cells at 26 weeks post treatment. (*e*) Flow Analysis of CD4^+^ T_CM_ and CD8^+^ T_CM_ cells in peripheral blood, demonstrating an increase in the percentage of CD4^+^ T_CM_ cells at 18 weeks post treatment. (*f*) Flow Analysis of CD4^+^ T_EM_ and CD8^+^ T_EM_ cells in peripheral blood, demonstrating a decline in the percentage of CD4^+^ T_EM_ and CD8^+^ T_EM_ cells at 18 weeks and 26 weeks respectively post treatment. (*g*) Flow Analysis of CD4^+^ HLA-DR^+^ in peripheral blood, demonstrating a decline in their percentages at 26 weeks post treatment. (*h*) Flow Analysis of CD8^+^ HLA-DR^+^ T cells in peripheral blood, demonstrating a decline in their percentages at 26 weeks post treatment.

To evaluate the immune modulating effects of the SCE therapy, we used flow cytometry to examine immune markers in 15 participants following SCE therapy. Clinical data indicated no changes in total cell numbers of each cell population during one-year follow-up, including leukocyte common antigen CD45^+^ nuclearized cells, CD3^+^ T cells, CD4^+^ T cells, CD8^+^ T cells, CD56^+^ NK cells, and CD20^+^ B cells (Fig. 3a). Quantification of the percentages of total CD4^+^ and CD8^+^ T cells in peripheral blood remained very stable over a year (Fig. 3b). Next, we explored the modulation of SCE therapy on different T-cell subpopulations by using the common surface markers for characterization of the naïve and memory T cells, such as CD45RA and CCR7 (Maecker et al., 2012) (Fig. 3c). Notably, the percentage of naïve CD4^+^ T (CD45RA^+^ CCR7^+^) cells was significantly increased at 26 weeks after the treatment with SCE therapy (41.1 ± 12.5 versus 32.6 ± 11.2 at baseline, *P* = 0.0042), and maintained through the final follow-up (44.3 ± 8.5 at 56 weeks post-treatment versus the baseline level, *P* = 0.0021) (Fig. 3d). The percentage of naïve CD8^+^ T cells did not exhibit significant changes at any follow-ups (Fig. 3d) (30.74 ± 8.54 at 56 weeks post-treatment versus 26.62 ± 14.2 at baseline, *P* = 0.35). These findings suggested that the SCE therapy restored the regeneration of naïve CD4^+^ T cells, an essential part of normal immune capacity.

To explore the effects of the SCE therapy on the memory T cells, T_CM_ and T_EM_ were examined by flow cytometry. Overall analysis in these subjects demonstrated that the percentage of CD4^+^ T_CM_ (CD45RA^−^ CCR7^+^) cells was markedly and constantly increased after receiving SCE therapy at 18 weeks (38.71 ± 11.7 versus 26.5 ± 15.4 at baseline, *P* = 0.018) (Fig. 3e). In contrast, the percentage of CD8^+^ T_CM_ cells was only temporarily improved at 18 weeks (16.78 ± 10.5 versus 9.8 ± 8.8 at baseline, *P* = 0.034), but return to baseline levels during continued follow-ups (Fig. 3e). In comparison with Group B subjects (4/9, 44%), Group A subjects (4/6, 67%) were more efficiently increasing the percentage of CD4^+^ T_CM_ cells over 30% of positive cells at 18 weeks follow-up (data not shown). Notably, overall analysis of T_EM_ (CD45RA^−^ CCR7^−^) cells revealed that both CD4^+^ T_EM_ cells and CD8^+^ T_EM_ cells were considerably decreased at 18 weeks (22.88 ± 9.4 versus 34.66 ± 18.48 at baseline, *P* = 0.03) and 26 weeks (20.2 ± 8.8 versus 29.34 ± 11.2 at baseline, *P* = 0.015) respectively (Fig. 3f). Group A subjects (6/6, 100%) were more efficiently decreasing the percentage of CD8^+^ T_EM_ cells over 15% of positive cells than that in Group B subjects (7/9, 78%) at 26 weeks follow-up; 5/6 (83%) of Group A subjects vs 7/9 (78%) of Group B subjects for the reduction of CD4^+^ T_EM_ cells at 26 weeks follow-up (data not shown).

In addition, using HLA-DR as an activation marker for T cells (Maecker et al., 2012), clinical data demonstrated that the percentage of CD4^+^ HLA-DR^+^ T cells and CD8^+^ HLA-DR^+^ T cells were markedly declined at 26 weeks follow-up relative to the baseline levels (Fig. 3g and h).

### 3.3 Up-regulation of CCR7 Expression on T Cells After Receiving SCE Therapy in T1D Subjects

**Fig. 4:**
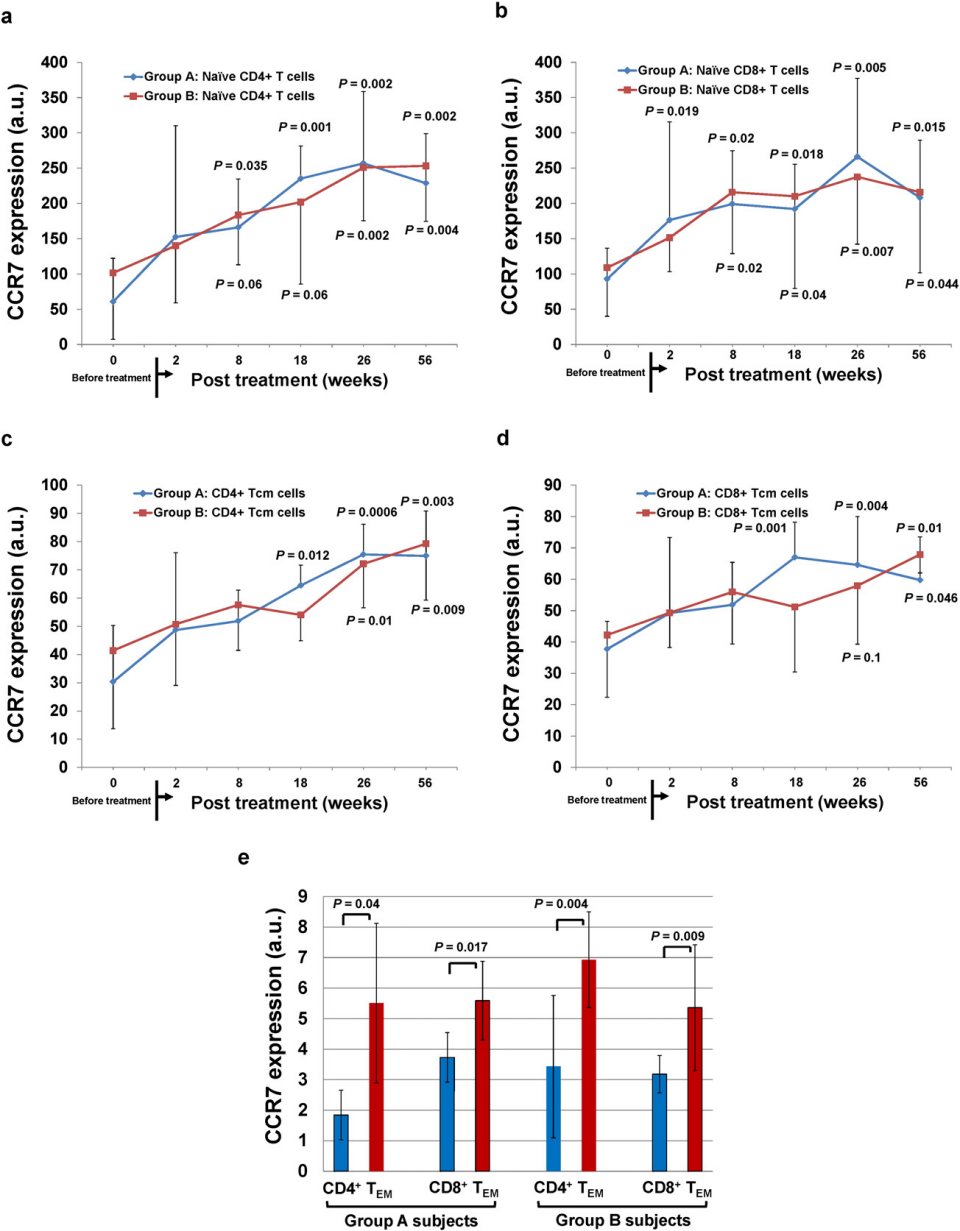
*Up-regulation of CCR7 expression on T cells in Caucasian T1D patients after SCE therapy*. All subjects received two treatments with SCE therapy. After 3 months, all subjects received a 2nd treatment with SCE therapy. Follow-up visits were scheduled 2, 8, 12, 18, 26, 40 and 56 weeks after treatment for clinical assessments and laboratory tests. Patient lymphocytes were isolated from peripheral blood by Ficoll-Hypaque (γ = 1.077) for flow cytometry analyses in T1D patients at baseline and different time points after SCE therapy. Isotype-matched IgG served as control. The levels of CCR7 expression were analyzed by Kaluza Flow Cytometry Analysis Software and present as arbitrary unit (a.u.). (*a*) Up-regulation of CCR7 expression on Naïve CD4^+^ T cells. (*b*) Up-regulation of CCR7 expression on Naïve CD8^+^ T cells. (*c*) Up-regulation of CCR7 expression on CD4^+^ T_CM_ cells. (*d*) Up-regulation of CCR7 expression on CD8^+^ T_CM_ cells. (*e*) Modulation of CCR7 expression on CD4^+^ and CD8^+^ T_EM_ cells. Data are shown as mean ± SD for all statistical analyses (*a*–*e*), paired Student's t test (*a*–*e*).

The C–C chemokine receptor 7 (CCR7) plays important roles in lymph-node homing of T cells via high endothelial venules and mediating the T-cell homeostasis (Forster et al., 2008). To further explore the modulation of SCE therapy, we analyzed the level of CCR7 expression on naïve T, T_CM_, and T_EM_ cells by flow cytometry. Clinical data revealed that both Group A and B subjects significantly increased the expression of CCR7 on Naïve CD4^+^ T cells (Fig. 4a), naïve CD8^+^ T cells (Fig. 4b), and CD4^+^ T_CM_ cells (Fig. 4c). The marked responses of Naïve CD4^+^ T cells in Group A subjects happened as early as at 8 weeks post SCE therapy (166.08 ± 68.81 at 8 weeks post-treatment versus 60.72 ± 61.62 at baseline, *P* = 0.035), comparable to that of delayed responses in Group B subjects at 26 weeks (251.04 ± 75.71 versus 101.59 ± 94.32 at baseline, *P* = 0.002) (Fig. 4a). The up-regulation of CCR7 expression on naïve CD8^+^ T cells was shown simultaneously at 8 weeks follow-up in both Group A and B subjects (Group A: 176.36 ± 139.14 at 2 weeks post-treatment versus 92.7 ± 43.98 at baseline, *P* = 0.019; Group B: 215.91 ± 87.06 at 8 weeks post-treatment versus 108.95 ± 68.94 at baseline, *P* = 0.02) (Fig. 4b). The expression of CCR7 on CD8^+^ T_CM_ cells in Group A subjects was also improved and started at 18 weeks follow-up (67.91 ± 11.24 versus 37.33 ± 8.88 at baseline, *P* = 0.001) (Fig. 4d), but with a postponed response in Group B subjects at 56 weeks follow-up (64.89 ± 5.86 versus 42.2 ± 19.83 at baseline, *P* = 0.046) (Fig. 4d). The levels (mean fluorescence intensity) of CCR7 expression on both CD4^+^ T_EM_ and CD8^+^ T_EM_ cells were markedly enhanced at 56 weeks follow-up after receiving SCE therapy in both groups (Fig. 4e). The data suggest that the up-regulation of CCR7 expression on CD4^+^ and CD8^+^ T cells may lead to the re-distribution of T cells in T1D subjects after the treatment with SCE therapy.

### 3.4 Up-regulation of CCR7 Expression on T Cells by ex Vivo Studies After the Treatment with CB-SCs

**Fig. 5:**
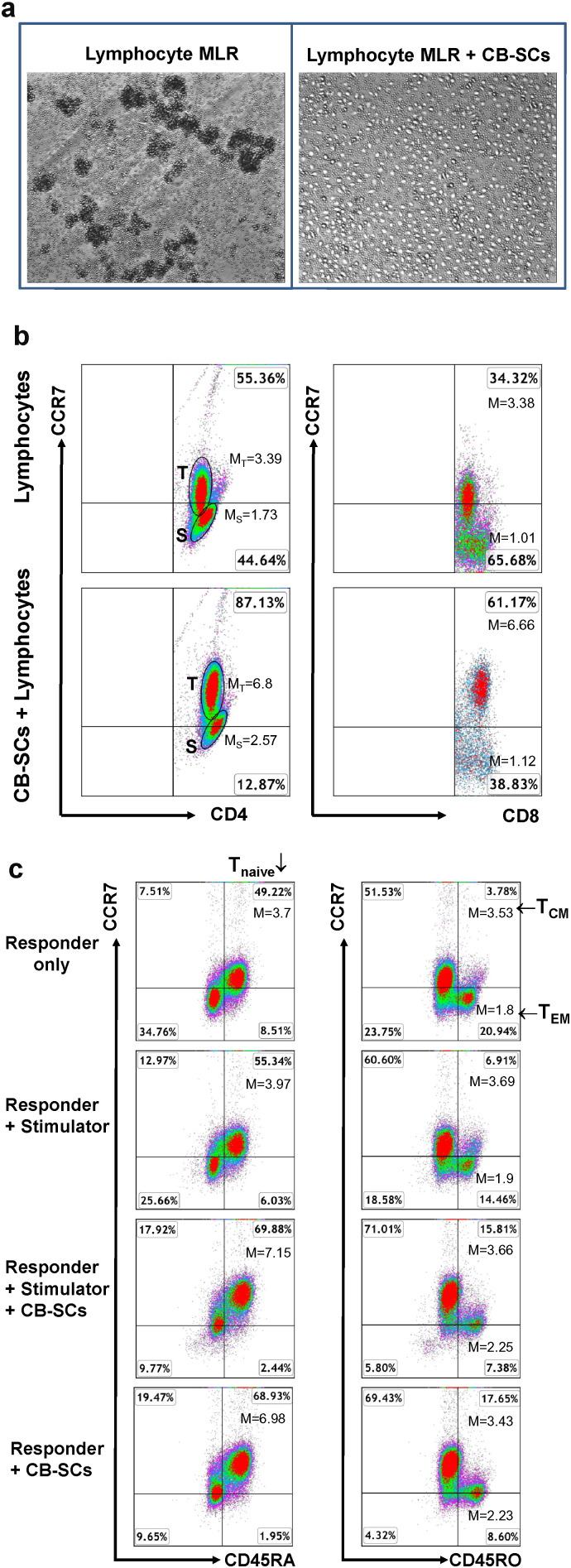
*Confirm the up-regulation of CCR7 expression on T cells by the ex vivo studies*. (*a*) Phase contrast microscopy shows the formation of cell clusters with different sizes in the mixed leukocyte reactions (MLR), in absence (left panel) of CB-SCs, but disappeared in presence (right panel) of CB-SCs. (*b* and *c*) Cells from the mixed leukocyte reactions were collected for flow analysis after co-culture for 5 days. Responder cells (R) were co-cultured with allogeneic stimulator cells (S) in the presence of CB-SCs. The ratio of R:S was 1:2; the ratio of CB-SCs:R was 1:10. (*b*) Flow cytometry of CCR7 expression on the gated CD4^+^ T cells and CD8^+^ T cells. The untreated CD4^+^ lymphocytes showed two populations: one was positive for CCR7 expression; another was negative (or very dim) for CCR7 expression (Top left panel). The mean fluorescence intensities of both populations were increased after treatment with CB-SCs (bottom left panel). (*c*) Flow cytometry of CCR7 expression on Naïve CD4^+^ T cells, CD45RO^+^ CCR7^+^ T_CM_ and CD45RO^+^ CCR7^−^ T_EM_ in the gated CD4^+^ T cells. The data showed the increase of the percentage of Naïve CD4^+^ T cells and CD4^+^ T_CM_ in the presence of CB-SCs. The percentages of CD4^+^ T_EM_ were decreased after treatment with CB-SCs.

CCR7 is a critical marker for the characterization of different T-cell subpopulations, which expression can be modulated by multiple factors (Britschgi et al., 2008). To further demonstrate the up-regulation of CCR7 on naïve T, T_CM_ and T_EM_ cells, the mixed leukocyte reaction (MLR) was employed to in the presence or absence of CB-SCs. Phase-contrast microscopy revealed significant numbers of cell clusters of varying sizes floating in the supernatant in the absence of CB-SC (Fig. 5a, left panel), but not in the presence of CB-SC (Fig. 5a, right panel). This indicated the suppressive activity of CB-SCs on the proliferation of T cells. Flow cytometry showed that the overall levels of CCR7 expression on CD4^+^ and CD8^+^ T cells were increased after the treatment with CB-SCs (Fig. 5b). We further analyzed the CCR7 expressions on the gated CD4^+^ T cells. In comparison with CB-SC-untreated groups (responder only or responder + stimulator), both percentage and mean fluorescence intensity (MFI) of CCR7 expression were enhanced on naïve T (CD45RA^+^ CCR7^+^) in the CB-SC-treated groups (responder + stimulator + CB-SCs or responder + CB-SCs) (Fig. 5c, left panels). The percentage of T_CM_ cells (CD45RO^+^ CCR7^+^) was increased in the CB-SC-treated groups; By contrast, the percentage of T_EM_ cells (CD45RO^+^ CCR7^−^) was decreased in the CB-SC-treated groups (Fig. 5c, right panels). The mean fluorescence intensity of CCR7 expression on T_EM_ cells (CD45RO^+^ CCR7^−^) was up-regulated from 1.85 ± 0.07 in the CB-SC-untreated groups to 2.24 ± 0.01 in the CB-SC-treated groups (*P* = 0.017).

To further confirm the up-regulation of CCR7 expression on T_EM_ cells, another major lymph node homing receptor CD62L was applied as a marker for human T_EM_ cells (CD45RO^+^ CD62L^−^) (Lefrancois, 2006, Unsoeld and Pircher, 2005). After treatment with CB-SCs, the level of CCR7 expression was analyzed on the gated CD45RO^+^ CD62L^−^ T_EM_ cells. The data demonstrated that the mean fluorescence intensity of CCR7 expression on CD45RO^+^ CD62L^−^ T_EM_ cells was upregulated from the baseline level 1.87 ± 0.04 to 2.09 ± 0.07 after the treatment with CB-SCs (*P* = 0.02). Therefore, these data suggested that the CCR7 expression on T_EM_ cells can be modulated by the treatment of CB-SCs.

### 3.5 Clinical Efficacy of SCE Therapy in the Improvement of Islet β Cell Function of T1D Subjects

**Fig. 6:**
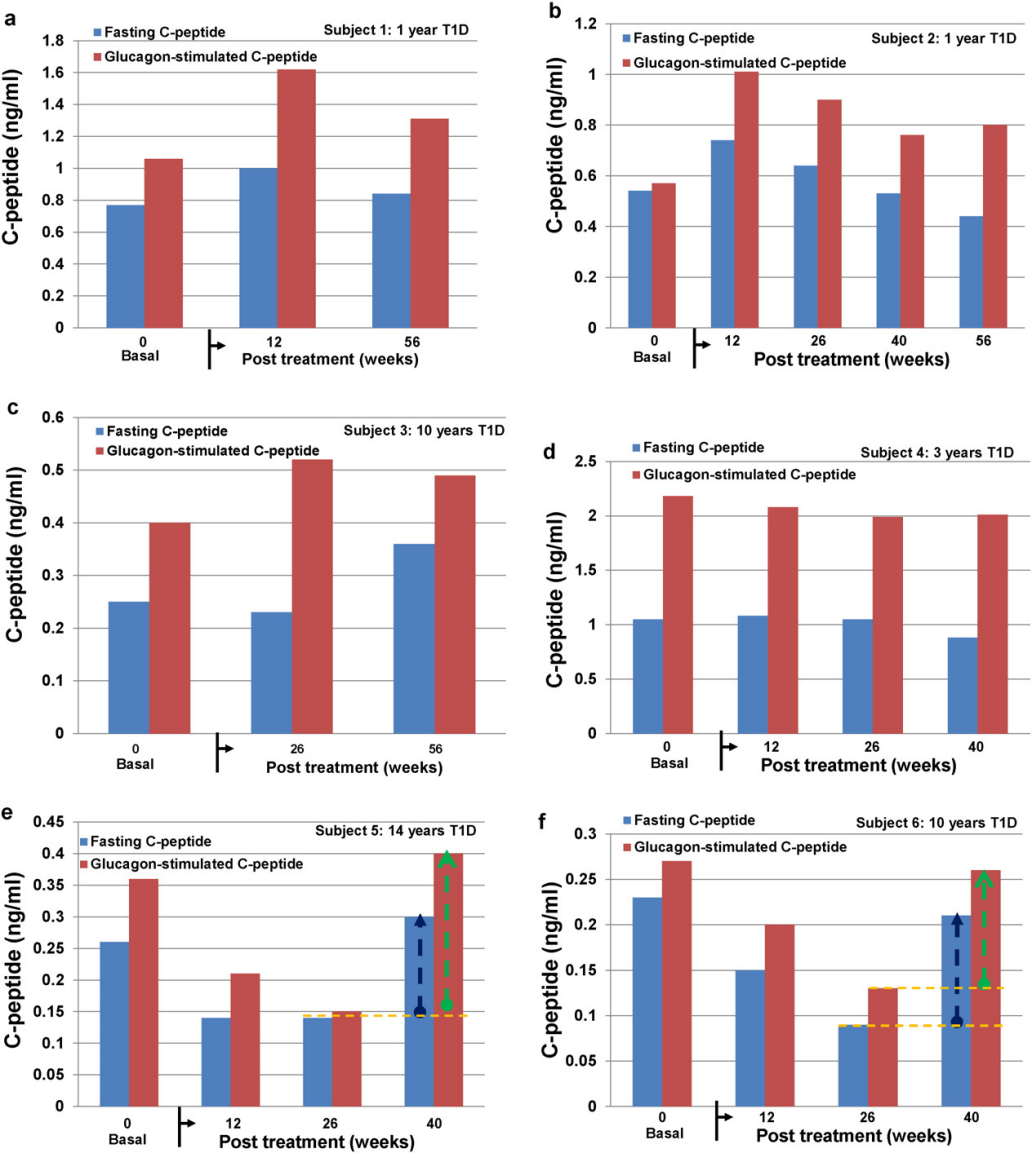
*Effects of SCE therapy on β-cell function in Caucasian T1D subjects*. All subjects received two treatments with SCE therapy (*a*–*f*). T1D subjects received two treatments with SCE therapy at the beginning and 3rd month respectively. Fasting (blue) and glucagon-stimulated C-peptide levels (brown) were examined at different time points according to the protocol. For glucagon-stimulated C-peptide production, glucagon (1 mg, i.v.) was administrated within 30 s, and six minutes later, plasma samples were collected for the C-peptide test by Ultrasensitive C-peptide ELISA kit. These data were from six T1D subjects with some residual islet β-cell function (Group A). (*a*–*d*) Recovered fasting and glucagon-stimulated C-peptide levels were retained in subject 1 through the final follow-up at 56 weeks post-treatments in subject 1–4 respectively. (*e* and *f*) show subjects 5 and 6 displayed some residual islet β-cell function beyond 10 years after diagnosis of T1D. After receiving SCE therapy, fasting C-peptide levels in Subject 5 initially decreased, but increased later at 40 weeks; fasting C-peptide levels in Subject 6 initially declined to 0.09 ng/ml at 26 weeks but improved to 0.21 ng/ml at 40 weeks. Their glucagon-stimulated C-peptide levels showed the similar tendencies as the fasting C-peptide levels.

To test the therapeutic potential of SCE therapy in the metabolic control of Caucasian T1D subjects, islet β-cell function was examined through the measurement of fasting plasma C-peptide and glucagon-stimulated C-peptide levels. In Group A subjects, clinical results demonstrated up-regulation of both fasting and glucagon-stimulated C-peptide levels at 12 weeks in two recent-onset T1D subjects (i.e., those most likely to have residual β cell populations) (Fig. 6a and b). Recovered fasting and glucagon-stimulated C-peptide levels were retained in subject 1 through the final follow-up at 56 weeks post-treatments (Fig. 6a). Glucagon-stimulated C-peptide levels in subject 2 were stable during one-year follow-up, while fasting C-peptide levels declined slightly (Fig. 6b). Subject 3 who had T1D 10 years at the time of study, still achieved modest improvements including an increase in fasting C-peptide from 0.25 ng/ml at basal to 0.36 ng/ml at 56 weeks and an increase in glucagon-stimulated C-peptide level from 0.4 ng/ml at basal to 0.52 ng/ml at 26 weeks (Fig. 6c). Subject 4 who had T1D 3 years at the time of the study, retained normal β-cell function with no significant change over time in fasting C-peptide levels from 1.05 ng/ml at baseline to 0.88 ng/ml at 40 weeks and in glucagon-stimulated C-peptide levels from 2.18 ng/ml at baseline to 2.01 ng/ml at 40 weeks (Fig. 6d). Interestingly, we found both subjects 5 and 6 displayed some residual islet β-cell function beyond 10 years after diagnosis of T1D. After receiving SCE therapy, fasting C-peptide levels in Subject 5 initially decreased from 0.23 ng/ml at baseline to 0.14 ng/ml at 26 weeks but increased to 0.3 ng/ml at 40 weeks (Fig. 6e); fasting C-peptide levels in Subject 6 initially declined from 0.26 ng/ml at baseline to 0.09 ng/ml at 26 weeks but improved to 0.21 ng/ml at 40 weeks (Fig. 6f). Their glucagon-stimulated C-peptide levels showed the similar tendencies as the fasting C-peptide levels. In summary, participants in Group A (that is, subjects with some residual islet β cell function) maintained their fasting C-peptide levels at 56 weeks post-treatment (0.46 ± 0.33 ng/ml versus 0.52 ± 0.34 ng/ml at baseline, *P* = 0.78) (Table 2). Consistently, the median daily doses of insulin (0.37 ± 0.16 U/kg body weight versus 0.38 ± 0.16 at baseline, *P* = 0.84) and the median glycated hemoglobin (HbA_1_C) (7.8 ± 1.48 versus 7.6 ± 1.3 at baseline, *P* = 0.81) were stabilized after 56 weeks post-treatment (Table 3). The data indicate that the residual β cell function in Group A patients was rescued and preserved after receiving SCE therapy, without a significant linear decline as the natural history of T1D (Battaglia and Atkinson, 2015).

Additionally, no changes were observed in fasting C-peptide levels of severe long-standing Group B patients with no residual pancreatic islet β cell function after receiving two SCE therapies (Table 2, Table 3). Their responses to SCE therapy were strikingly different from that reported in long-standing severe Chinese T1D subjects (Zhao et al., 2012, Zhao, 2012). The potential mechanisms underlying this difference need to be explored.

## 4 Discussion

Overcoming autoimmune memory is essential for eliminating autoimmunity in T1D and other autoimmune diseases. The current studies demonstrated the safety and feasibility of a two-treatment approach with SCE therapy, without significantly changing the numbers and ratios of different cell compartments in the subjects' immune system. Both the percentage of CD4^+^ T_EM_ and CD8^+^ T_EM_ cells were substantially decreased in the peripheral blood of these Caucasian T1D subjects of European background after receiving SCE therapy, whereas the CD4^+^ T_CM_ appeared to be favored by SCE therapy. Notably, the levels of CCR7 expression on naïve T and T_CM_ cells were markedly increased after SCE therapy, further confirmed by ex vivo studies. The percentage of CCR7^+^ T_CM_ increased at the expense of CCR7^−^ T_EM_. These findings provide a novel solution to alter the autoimmune memory compartment in T1D.

Naïve T cells constantly recirculate between secondary lymphoid tissue (SLT) using the blood and lymph as conduits. The present study revealed that the percentage of naïve CD4^+^ T cells was markedly increased in T1D subjects after receiving SCE therapy. The number of total CD4^+^ T cells was constantly maintained in peripheral blood during one-year follow-up. Due to the short lifespan (3 months) for most T cells, the data suggest that the expansion of naïve CD4^+^ T cells represented a normal restoration of immune system balance to the T1D subjects by SCE therapy. Clinical evidence demonstrates that population of T_EM_ is increased in chronic inflammation or autoimmune diseases, such as chronic rhinosinusitis (Pant et al., 2013) and long-standing T1D subjects (Matteucci et al., 2011). Additionally, both the percentage and absolute cell number of naïve T cells and T_CM_ were reduced in long-standing T1D subjects (Matteucci et al., 2011). Notably, the present clinical data demonstrated that the percentage of Naïve CD4^+^ T cells and T_CM_ were all significantly increased, but CD4^+^ T_EM_ and CD8^+^ T_EM_ declined in these T1D subjects after receiving SCE therapy. Thus, the data demonstrate that SCE therapy corrected the dysfunction of T_EM_ and favored the differentiation of T_CM_ in long-standing T1D subjects. Differently, both CD4^+^ T_CM_ and T_EM_, together with CD8^+^ T_CM_ (but not CD8^+^ T_EM_), were all decreased by the treatment of new-onset T1D patients with Alefacept therapy in T1DAL trial, an approach that use the genetically engineered fusion protein targeting and deleting CD2^+^ T cells (Rigby et al., 2013).

**Fig. 7:**
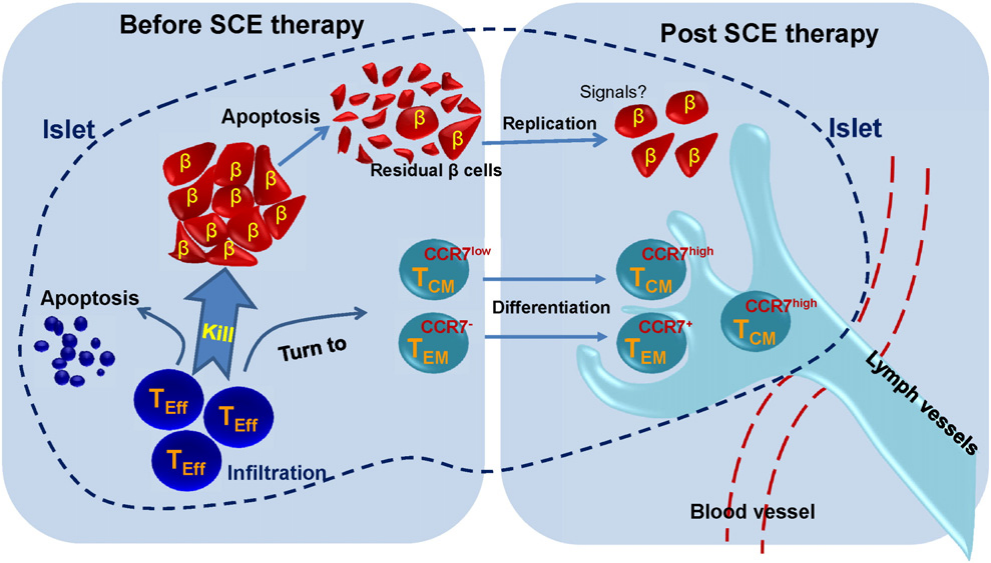
*Proposed model for the molecular and cellular mechanisms underlying SCE therapy for the treatment of T1D*. The up-regulation of CCR7 expression on CD4^+^ T_CM_, CD8^+^ T_CM_, CD4^+^ T_EM_, and CD8^+^ T_EM_ cells after receiving SCE therapy (right panel) may lead to the evacuation of these infiltrated autoimmune cells (left panel) from insulitic lesions through the draining of lymphatic vessels in pancreatic islets (dashed line) of T1D subjects. This restoration of homeostasis in pancreatic islets may result in the regeneration of islet β cells via potential signaling pathways.

Insulitis is the predominant pathological feature in pancreatic islets of T1D subjects caused by the infiltration of autoimmune CD8^+^ and CD4^+^ T cells, CD20^+^ B cells, and macrophages (Campbell-Thompson et al., 2013, In't, 2011). T_CM_ cells trafficking in peripheral blood maintain the capacity to home to lymph nodes due to retained expression of CCR7 and CD62L molecules, which are required for migration from blood into lymph nodes across the high endothelial venules. Notably, current study revealed the modulation of CCR7 expression on CD4^+^ T_CM_, CD8^+^ T_CM_, CD4^+^ T_EM_, and CD8^+^ T_EM_ cells after receiving SCE therapy in T1D subjects. These outcomes may lead to the evacuation of autoimmune cells from insulitic lesions through lymphatic vessels which express CCL19 and CCL21, the two ligands of CCR7 (Fig. 7). Additionally, the improvement of CCR7 expression on Naïve CD4^+^ and naïve CD8^+^ T cells may contribute to the redistribution and polarization of T cells and result in the restoration of homeostasis in immune system. Thus, SCE therapy delivers not only the recovery of homeostasis in pancreata, but also the comprehensive immune balance for the whole body.

In comparison with conventional immune therapies (e.g., monoclonal antibodies, vaccines, Treg therapy, and dendritic cell therapy) for T1D, the ex vivo immune modulation by CB-SCs inside a device can be controlled and monitored during the treatment. The lymphocytes (including Naïve T cells, T_CM_, and T_EM_) purified by apheresis can be intensively educated by directly contact with CB-SCs, with minimum interference from red blood cells, granulocytes, and other blood components. This approach also reduces side effects associated with conventional immune therapies. Based on our combined preclinical (Zhao et al., 2007, Zhao et al., 2009, Zhao and Mazzone, 2010) and clinical studies (Zhao et al., 2012, Zhao et al., 2013) to date, immune modulation by CB-SCs seems to be mediated by a variety of molecular and cellular mechanisms including: *1*) Expression of autoimmune regulator (AIRE) in CB-SCs plays an essential role (Zhao et al., 2012); *2*) Functioning via cell–cell contact mechanisms involving the surface molecule programmed death ligand 1 (PD-L1) (Zhao et al., 2007) and CD270 on CB-SCs, and their ligands PD-1 and BTLA on variety of immune cells (e.g., T cells, B cells, monocytes, dendritic cells, and granulocytes (Li et al., 2015); *3*) Acting through soluble factors released by CB-SCs (e.g., nitric oxide, TGF-β1) (Zhao et al., 2007); and *4*) Adjusting the cell–cell interaction between antigen-presenting cells monocytes/macrophages and T cells through co-stimulating molecules and their ligands (Zhao et al., 2013). Thus, during the ex-vivo brief exposure to CB-SCs, T1D-derived T_CM_ and T_EM_ can be “educated” by the favorable microenvironment created by CB-SCs through cell to cell contact and soluble factors.

Previous work demonstrated that a single treatment with SCE therapy can significantly improve fasting C-peptide levels, reduce the median glycated hemoglobin A_1_C (HbA_1_C) values, and dramatically decrease the median daily dose of insulin in Chinese patients with some residual β cell function and patients with no residual pancreatic islet β cell function (Zhao et al., 2012). Consistently, we found the regeneration of islet β cells in SCE-treated long-standing Chinese T2D subjects (Zhao et al., 2013), which express a shortage of islet β cells similar to T1D subjects. The current study demonstrated up-regulation of fasting and glucagon-stimulated C-peptide levels in Caucasian T1D subjects with some residual β cell function, including subjects with longstanding T1D. This response to SCE therapy was similar in Chinese T1D subjects, but the long-standing severe Caucasian T1D subjects with no residual pancreatic islet β cell function failed to show any increase in fasting C-peptide. To improve its clinical efficacy, it will be essential to clarify the molecular and cellular mechanisms underlying these different responses to the SCE therapy. Ongoing mechanistic studies revealed the molecular disparities of expression of muscarinic acetylcholine receptors (mAChRs) on human islet β-cell regeneration between Caucasian and Chinese populations (Figs. S3 and S4). The M2 receptor and other specific cholinergic markers such as vesicular acetylcholine transporter (vAChT) and choline acetyltransferase (ChAT) are substantially expressed on the islet β cells of the Chinese population (Fig. S3). It indicates the autocrine cholinergic signal involved in the functional regulation of islet β cells of Chinese population. However, in pancreata of Caucasian population, M3 and M5 muscarinic receptors were strongly expressed on the islet β cells (Molina et al., 2014); pancreatic islet α cells display M2 receptor and produce cholinergic signal that contributes to the modulation of islet β-cell function via the paracrine pathway (Rodriguez-Diaz et al., 2011). Additionally, animal studies have shown that parasympathetic innervation modulates the β-cell proliferation and function of pancreatic islets (Lausier et al., 2010, Rodriguez-Diaz et al., 2012), suggesting a possible mechanism for differences in response to SCE therapy based on the different receptor subtypes. The M2 receptor-mediated signaling may function as a new molecular target that contribute to the modulation of islet β-cell expansion.

Controlling the immune dysfunctions and overcoming the shortage of islet β cells are two critical steps for the treatment of T1D. Conventional immunotherapies (e.g., CD3 and CD20 mAb treatments) targeting the general immune cell compartment usually leads to the absolute decline of cell numbers due to their broad cytotoxicity. These approaches may make patients more vulnerable to pathogens and certainly raise concerns about clinical safety and tolerability. SCE therapy modifies rather than destroys the cells responsible for autoimmune responses. Current clinical data demonstrated the safety and efficacy of the SCE therapy for the immune modulation of Caucasian T1D subjects of European background. Due to the plasticity of human T cells, the induction of differentiation of T cells into different functional subsets and the correction of functional defects of T_EM_ can be efficiently achieved through SCE therapy in T1D subjects. SCE therapy provides lasting reversal of autoimmunity that allows the native improvement of islet β-cell function in T1D subjects with residual β cell function. A better understanding of the molecular disparities of islet β cells between Caucasian and Chinese populations will provide insight into possible methods for improving treatment efficacy and β cell recovery in Caucasians with T1D who do not have residual β cell function prior to treatment. SCE therapy may revolutionize the clinical treatment of diabetes without the safety and ethical concerns associated with conventional approaches.

## Competing Interests

Dr. Zhao, an inventor of Stem Cell Educator technology, led the clinical study, and has an investment and a fiduciary role in Tianhe Stem Cell Biotechnology Inc. (licensed this technology from University of Illinois at Chicago). All other authors (ED, MP, BS, HZ, EMR, JG, SP, MA, EM, CL, RT, ZZ, WH, TM, EG, and JO) have no financial interests that may be relevant to the submitted work.

## Authors' Contributions

YZ, JO and ED designed the trial and analyzed the data. YZ drafted the manuscript. YZ and JO obtained the funding. YZ, ED, MP, and BS collected data for analysis and interpretation. HZ, EMR, JG, SP, MA, EM, CL, RT, ZZ, HW, TM, and EG were for data interpretation, administrative, technical, or material support. All authors read and approved the final manuscript.

## Figures and Tables

**Table 1:** Characteristics of Caucasian T1D subjects before treatment.

Patient No.	Age	Gender	History (year)	Fasting C-peptide (ng/ml)	Post-glucagon C-peptide (ng/ml)	HbA_1_C (%)	Insulin dose (U/kg body weight)
*Group A: T1D patients with residual islet β cell function*							
1	36	M	1	0.77	1.06	7.1	0.28
2	27	F	1	0.54	0.57	6.3	0.32
3	31	F	10	0.25	0.4	9	0.52
4	20	M	3	1.05	2.18	6.2	0.18
5	37	M	14	0.26	0.36	7.8	0.4
6	52	M	10	0.23	0.27	9.2	0.61
*Mean (SD)*	*33.8 (10.9)*		*6.5 (5.5)*	*0.52 (0.34)*	*0.81 (0.73)*	*7.6 (1.3)*	*0.38 (0.16)*

*Group B: T1D patients with no residual islet β cell function*							
7	30	M	23	< 0.01	< 0.01	7.1	0.62
8	37	M	15	< 0.01	< 0.01	6.2	0.72
9	40	F	12	< 0.01	< 0.01	8.4	0.92
10	53	F	14	< 0.01	< 0.01	9.1	0.79
11	33	M	13	< 0.01	< 0.01	7.4	0.56
12	48	F	6	< 0.01	0.04	10.1	0.58
13	32	M	6	< 0.01	0.01	8.3	0.51
14	45	M	17	< 0.01	< 0.01	6.5	0.71
15	45	M	6	0.02	0.06	8.4	0.96
*Mean (SD)*	*40.3 (7.9)*		*12.4 (5.8)*	*0.01 (0.003)*	*0.02 (0.02)*	*7.9 (1.3)*	*0.71 (0.16)*

**Table 2:** Changes in C-peptide levels of the T1D subjects after treatment at 12 months.

Patient No.	Before treatment			12 m after SCE therapy		
Basal glycemia	Basal C-peptide	Post-glucagon C-peptide	Basal glycemia	Basal C-peptide	Post-glucagon C-Peptide
*Group A: T1D patients with residual islet β cell function*						
1	100	0.77	1.06	175	0.84	1.31
2	85	0.54	0.57	149	0.44	0.8
3	155	0.25	0.4	280	0.36	0.49
4	141	1.05	2.18	130	0.88	2.01
5	144	0.26	0.36	178	0.17	0.23
6	218	0.23	0.27	102	0.08	0.1
*Mean*	*140.5*	*0.52*	*0.81*	*169*	*0.46*	*0.82*
*(SD)*	*46.8*	*0.34*	*0.73*	*61.4*	*0.33*	*0.73*

*Group B: T1D patients with no residual islet β cell function*						
7	230	< 0.01	< 0.01	232	0.01	0.01
8	128	< 0.01	< 0.01	135	0.01	0.01
9	144	< 0.01	< 0.01	244	0.01	0.01
10	198	< 0.01	< 0.01	173	0.01	0.01
11	211	< 0.01	< 0.01	182	0.01	0.01
12	111	< 0.01	0.04	195	0.01	0.01
13	165	< 0.01	0.01	174	0.01	0.02
14	69	< 0.01	< 0.01	123	0.01	0.01
15	243	0.02	0.06	151	0.02	0.01
*Mean*	*166.56*	*< 0.01*	*0.037*	*178.78*	*0.01*	*0.01*
*(SD)*	*58.56*		*0.025*	*40.64*	*0.003*	*0.003*

**Table 3:** Changes in HbA_1_C levels and insulin doses of the T1D subjects after treatment at 12 months.

Patient No.	HbA_1_C		Insulin dose (U/kg body weight)	
Before treatment	12 m after SCE	Before treatment	12 m after SCE
*Group A: T1D patients with residual islet β cell function*				
1	7.1	7.4	0.28	0.29
2	6.3	6.3	0.32	0.30
3	9	9.3	0.52	0.52
4	6.2	5.9	0.18	0.19
5	7.8	8.9	0.4	0.3
6	9.2	9	0.61	0.6
*Mean*	*7.6*	*7.8*	*0.38*	*0.37*
*(SD)*	*1.3*	*1.48*	*0.16*	*0.16*

*Group B: T1D patients with no residual islet β cell function*				
7	7.1	7.5	0.62	0.63
8	6.2	6	0.72	0.74
9	8.4	7.9	0.92	0.89
10	9.1	8.4	0.79	0.8
11	7.4	7.5	0.56	0.56
12	10.1	9.2	0.58	0.57
13	8.3	6.8	0.51	0.5
14	6.5	6.6	0.71	0.72
15	8.4	8.3	0.96	0.9
*Mean*	*7.94*	*7.58*	*0.71*	*0.7*
*(SD)*	*1.26*	*1*	*0.16*	*0.15*

## Appendix A Supplementary Data

Supplementary Data

Clinical Protocol
