# Supplementary material for: Modulation of Autoimmune T-Cell Memory by Stem Cell Educator Therapy: Phase 1/2 Clinical Trial
Source: eBioMedicine. 2015 Nov 5;2(12):2024–36. doi: 10.1016/j.ebiom.2015.11.003 (PMC4703710; doi:10.1016/j.ebiom.2015.11.003)
Supplement: Supplementary file 1 — Supplementary Data [file mmc1.pdf]

Supplementary Data:

1. Characterization of CB-SCs by flow cytometry

For the markers of CB-SCs, the leukocyte common antigen CD45 and embryonic stem (ES) cell-related transcription factor OCT3/4 are normally used for the characterization of CB-SCs by flow cytometry (**Figure S1**).

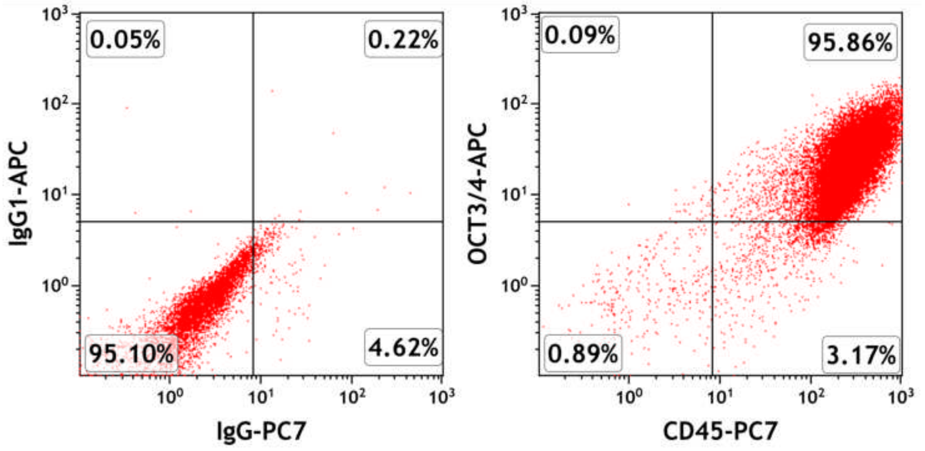

Figure S1. Flow cytometry. Analysis of CB-SCs with cellular markers such as leukocyte common antigen CD45 and embryonic stem cell marker OCT3/4. For immunostaining, cells were fixed and permeablized using a PerFix-nc kit (Beckman Coulter), and then stained with PC7-conjugated mouse anti-human CD45 mAb (Beckman Coulter) and Alexa Fluor 647-conjugated rat anti-human OCT 3/4 mAb (eBioscience, San Diego, CA) for 30 min at room temperature. Isotype-matched mouse anti-human IgG antibodies (Beckman Coulter) served as a negative control for all fluorescein-conjugated IgG mAbs.

2. *Ex vivo* studies demonstrate the fast modulation on T cells by the treatment with CB-SCs

To explore the modulation of CB-SCs on early T cell activation pathways, PBMCs were planted in SCE devices. After incubation for 3 hours, flow cytometry demonstrated the phosphorylation of tyrosine residues in proteins was markedly increased in SCE-treated lymphocytes compared to control lymphocytes (**Figure S2 a**), suggesting that SCE treatment acts very quickly on lymphocytes. The proportion of phosphorylated $CD4^+$ T cells was improved from 22·75% ± 1·24% of control lymphocytes to 35·61% ± 0·6% of SCE-treated lymphocytes

(P = 0·007); the percentage of phosphorylated CD8$^+$ T cells was improved from 32·99% ± 0·62% of control lymphocytes to 44·65% ± 2·7% of SCE-treated lymphocytes (P = 0·03) (**Figure S2 b**).

Figure S2. Detection of phosphor-tyrosine by flow cytometry. Lymphocytes were treated with SCE for 3 hours at room temperature in serum-free culture medium. Lymphocytes cultured in the absence of CB-SCs served as control. **(a)** Expression of phospho-tyrosine in SCE-treated lymphocytes (red line) was overlaid with that of SCE-untreated control lymphocytes (blue line). **(b)** Phospho-tyrosine levels in the gated CD4$^+$ and CD8$^+$ T cells.

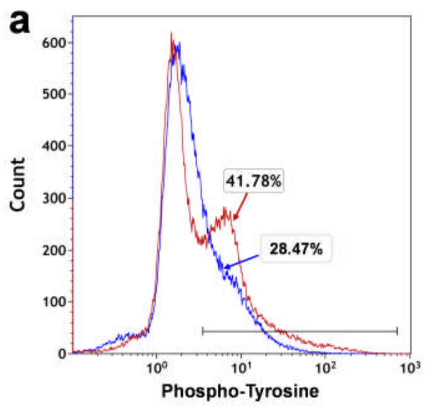

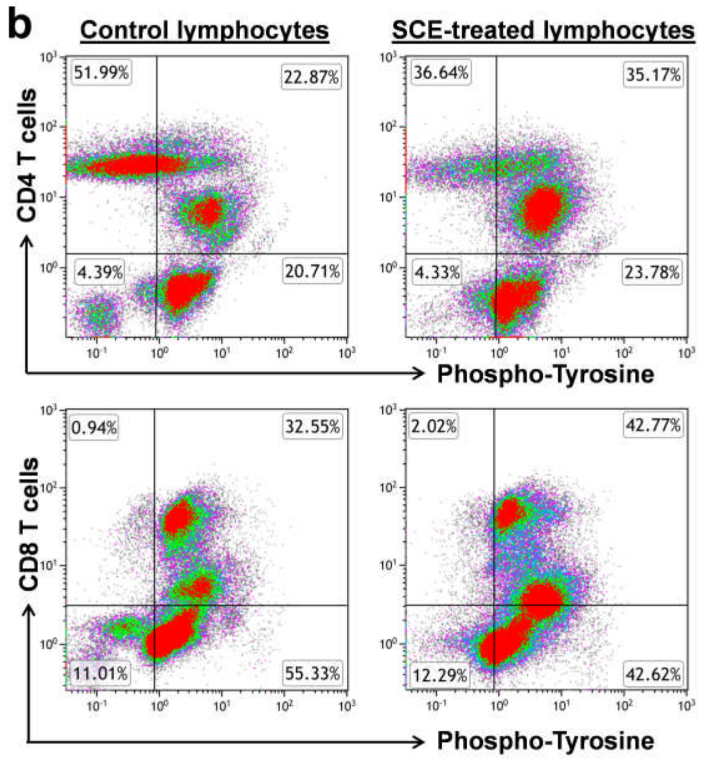

3. A marked disparity of muscarinic acetylcholine receptor M2 expression on islet β cells between Caucasian and Chinese populations.

To improve the clinical efficacy of SCE therapy and identify the molecular and cellular mechanisms underlying this different response between Caucasian and Chinese populations after receiving SCE therapy, immunohistochemical studies of pancreatic islets revealed a critical difference between Caucasian and Chinese islet β cells that may underlay the difference in response. Specifically, muscarinic acetylcholine receptor M2 (one of G_i/G_o-protein-coupled receptors) is strongly expressed on the islet β cells of the Chinese population but only very weakly or not expressed on the islet β cells of the Caucasian population (**Figures S3 a and b**). The expressions of other specific cholinergic neurotransmitter-associated markers such as vesicular acetylcholine transporter (vAChT) (**Figure S3 c**) and choline acetyltransferase (ChAT)

(**Figure S3 d**), as well as a neuronal marker protein gene product 9·5 (PGP 9·5) (**Figures S3 e and f**) are displayed in the islet β cells of the Chinese population. Interestingly, these markers were found only on the islet α cells of the Caucasian population (Rodriguez-Diaz et al., 2011). Triple immunohistochemistry demonstrated that the expression of vACht was found in both islet β and α cells of Chinese pancreata (**Figure S3 c**). Additional studies confirmed that there were very weak or negative expressions of M3, M4, and M5 receptors on the islet β cells of the Chinese population (**Figures S4 a - c**). To clarify the role of acetylcholine signaling involved in the regeneration of islet β cells, immunohistochemistry proved the co-localization of Ki67 (a proliferation marker) and vACht in Chinese islet β cells (**Figure S4 d and e**). Thus, the data demonstrated that molecular and cellular signatures underlying differences in islet β-cell regeneration between Caucasian and Chinese populations.

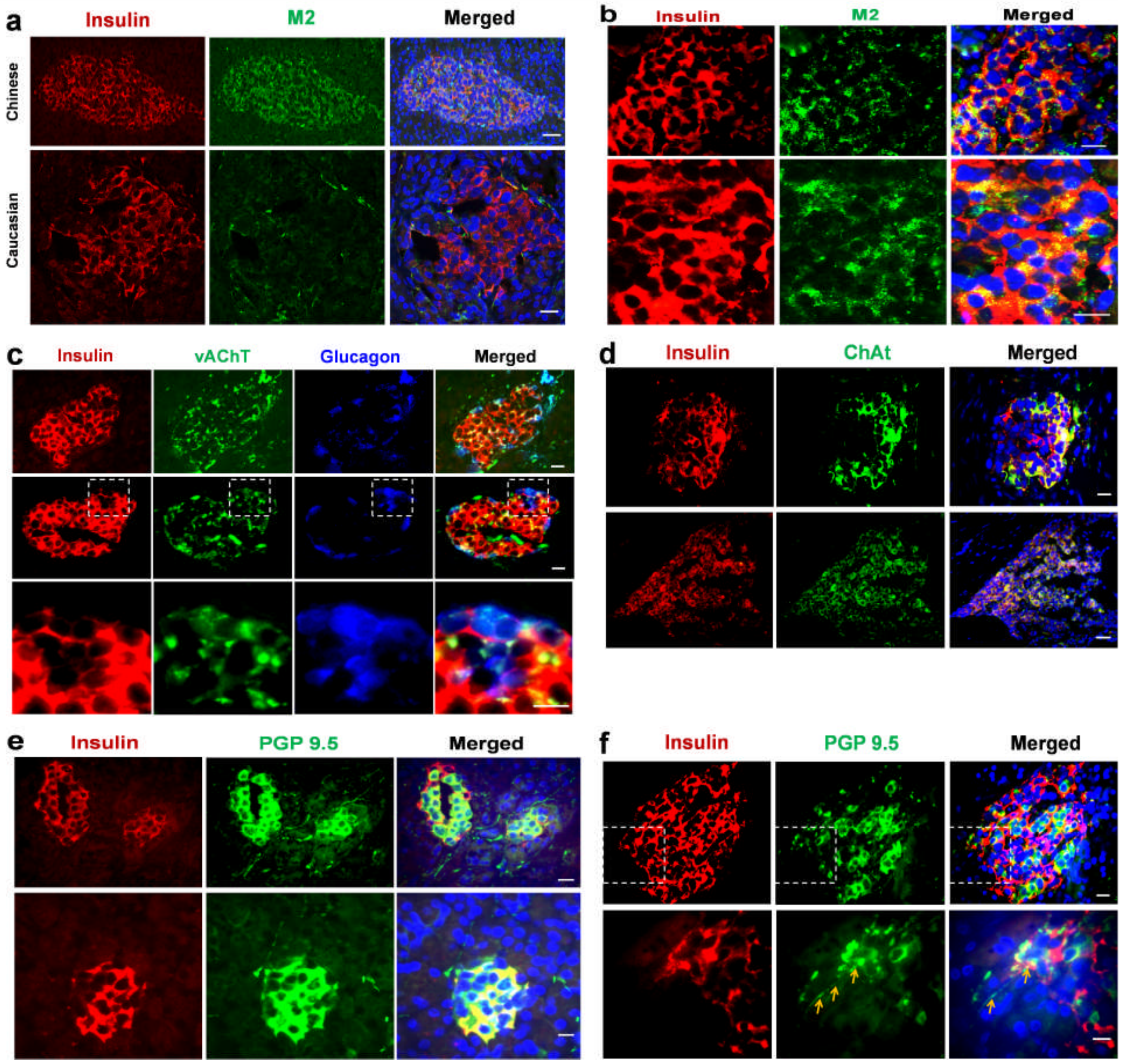

Figure S3. Characterization and comparison of expression of muscarinic acetylcholine receptor M2 on pancreatic islet β cells between Caucasian and Chinese populations. Images were acquired with a confocal microscope Nikon A1R confocal microscope and analyzed with NIS Elements Viewer 4.20 software. **(a)** Representative pictures of pancreatic islets shows the expression of M2 receptor (green) on the islet β cells (red) of Chinese population, but not on the islet β cells of Caucasian population (bottom panels). The merged image shows the co-localization in yellow. **(b)** At high magnification, the expression of M2 receptor (green) was localized on the islet β cells (red) of Chinese population. **(c)** Triple immunostainings show the expression of the cholinergic marker vACht (green) in islet β cells (red) and α cells (blue) of Chinese population. (d-f) Pancreatic islet β cells of Chinese populations display other cholinergic neuronal markers ChAt and PGP 9·5. **(d)** Representative images of pancreatic islets shows the expression of ChAt receptor (green) on the islet β cells (red) of Chinese population. **(e)** Expression of PGP 9·5 (green) in different islet β cells (red) of Chinese population. **(f)** The strong expression of PGP 9·5 (green) in Chinese pancreatic islet β cells (red). High magnifications in bottom panels show the innervation of a PGP 9·5-positive neuronal fiber (green, pointed by orange arrows) to pancreatic islet β cells (red). High magnification is shown in bottom panels. The experiments were independently performed five times for a and b and three times for c. The experiments were independently performed four times for d-f. Bars: 100 μm.

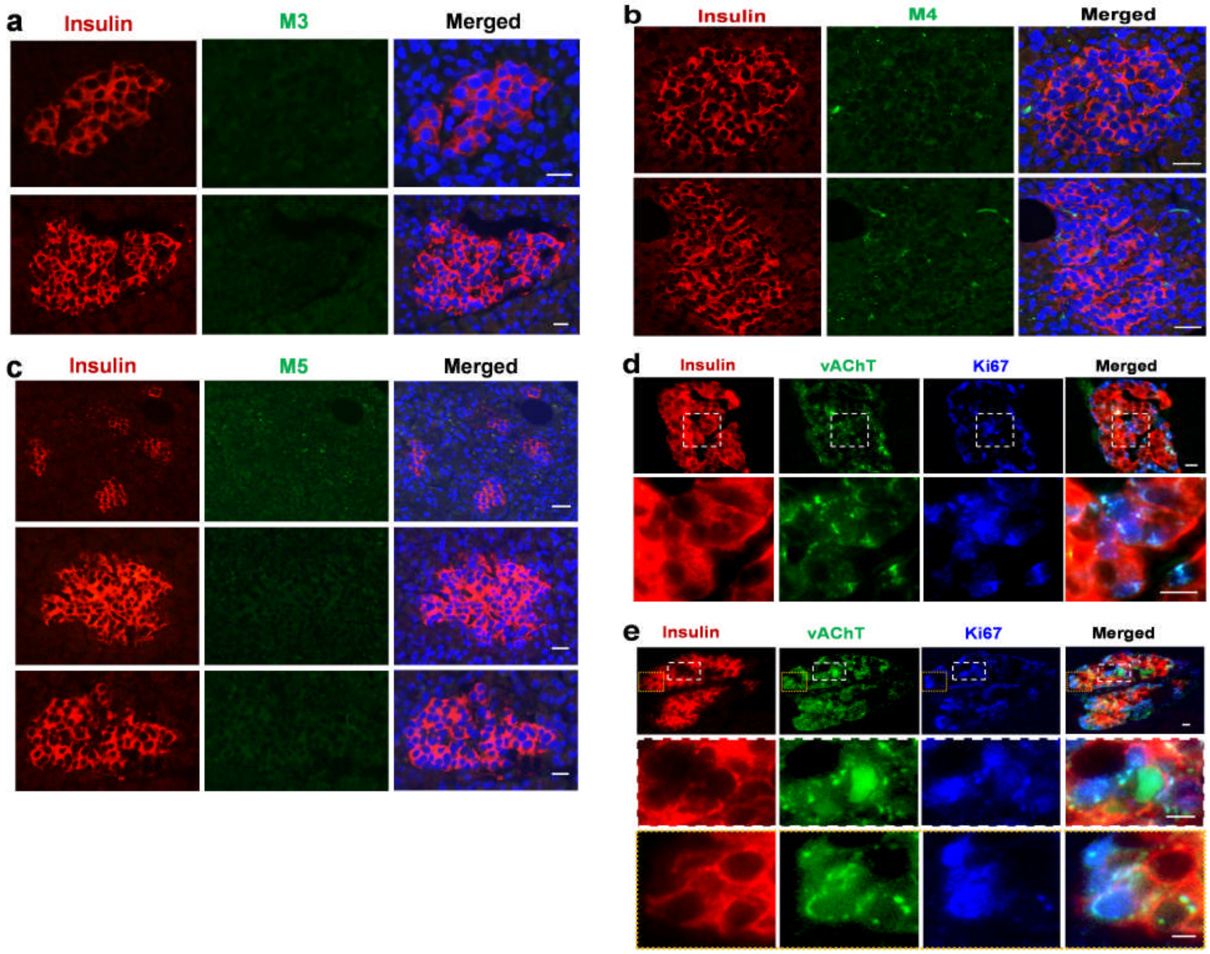

Figure S4. Immunohistochemistry testing the expression of M3, M4, and M5 receptors on pancreatic islet β cells of Chinese population. (a) Representative pictures show that Chinese pancreatic islet β cells (red) failed to display M3 receptor (green). **(b)** Confocal image of pancreatic islet β cells shows a weak expression of M4 receptor (green). **(c)** Expression of M5 receptor (green) at background level on pancreatic islet β cells (red) of Chinese population. (d, e) Pancreatic histology analyses reveal the colocalization of vAChT in the proliferated islet β cells. Pancreata from Chinese donors (n = 5) were collected for triple immunostaining with pancreatic islet β-cell marker (insulin, red), a cholinergic neuron-specific marker (vAChT, green), and a cell proliferation marker Ki67 (blue). **(d)** Immunohistochemistry shows that islet β cells (red) were positive for vAChT (green) and Ki67 (blue). High magnifications show the colocalization of vAChT and Ki67 in islet β cells (bottom panels). **(e)** Two pancreatic islets exhibit the colocalization of vAChT (green) and Ki67 (blue) in pancreatic islet β cells (red).The experiments were independently performed three times for a-c. Bars: 100 μm.

4. Methods for pancreatic immunohistochemistry

Pancreatic tissues were obtained from donors of Caucasian (n = 3) and Chinese populations (n = 5). The principal investigator received ethical approvals for the study protocol and consent form from Institutional Review Boards (IRBs). Pancreata were fixed in 10% formaldehyde, processed, and embedded in paraffin. Pancreatic sections were cut at 5 μm thickness. Immunohistochemistry was performed as previously described (Zhao et al., 2009). After

deparaffinization and rehydration through xylene and serial graded ethanol, pancreatic sections were treated with diluted Vector® Antigen Unmasking Solutions (Vector Laboratories, Burlingame, CA) according to manufacturer's protocol. To block non-specific staining, sections were incubated in a buffer containing 2·5% horse serum (Vector Laboratories) for 20 min at room temperature. Pancreatic sections were immunostained for 2 hrs at room temperature with different primary Abs including guinea pig polyclonal anti-insulin Ab (DakoCytomation, Carpinteria, CA), mouse anti-glucagon mAb (Sigma), mouse anti-muscarinic acetylcholine receptor M2 mAb (GeneTex, Irvine, CA), rabbit anti-muscarinic acetylcholine receptor M3 polyclonal Ab and rabbit anti-muscarinic acetylcholine receptor M5 polyclonal Ab (Abcam, Cambridge, MA), mouse anti-muscarinic acetylcholine receptor M4 mAbs, rabbit anti-vesicular acetylcholine transporter (vACht) polyclonal Ab, rabbit anti-protein gene product 9·5 (PGP 9·5), rabbit anti-choline acetyltransferase (ChAT) mouse anti-choline acetyltransferase mAb (EMD Millipore, Temecula, CA), DyLight405-conjugated and unconjugated rabbit anti-Ki67 Ab (Novus Biologicals USA, Littleton, CO). After washing with PBS, sections were incubated for 60 min at room temperature with second Abs included Cy3-conjugated AffiniPure donkey anti-guinea pig IgG, FITC-conjugated AffiniPure donkey anti-rabbit IgG, AMCA AffiniPure Donkey Anti-Rabbit IgG, FITC-conjugated AffiniPure donkey anti-mouse IgG, and AMCA-conjugated AffiniPure donkey anti-mouse IgG were purchased from Jackson ImmunoResearch Laboratories (West Grove, PA). For isotype-matched controls, mouse $IgG_{1\kappa}$ was purchased from BD Biosciences, guinea pig serum and rabbit IgG from Jackson ImmunoResearch Laboratories. After immunostaining, slides were mounted with VECTASHIELD Antifade Mounting Medium or VECTASHIELD Antifade Mounting Medium with DAPI (Vector Laboratories). After mounting, pancreatic slides were photographed with a Nikon A1R confocal microscope and analyzed by NIS Elements Viewer 4·20 software, or ZEISS Imager M1 equipped with AxioCam MRc camera and AxioCam MR Rev 3 software.

Reference List

Rodriguez-Diaz,R., Dando,R., Jacques-Silva,M.C., Fachado,A., Molina,J., Abdulreda,M.H., Ricordi,C., Roper,S.D., Berggren,P.O., and Caicedo,A. 2011. Alpha cells secrete acetylcholine as a non-neuronal paracrine signal priming beta cell function in humans. Nat. Med. 17, 888-892.

Zhao,Y., Lin,B., Darflinger,R., Zhang,Y., Holterman,M.J., and Skidgel,R.A. 2009. Human cord blood stem cell-modulated regulatory T lymphocytes reverse the autoimmune-caused type 1 diabetes in nonobese diabetic (NOD) mice. PLoS. ONE. 4, e4226.
