# Supplementary material for: Modulation of Autoimmune T-Cell Memory by Stem Cell Educator Therapy: Phase 1/2 Clinical Trial
Source: eBioMedicine. 2015 Nov 5;2(12):2024–36. doi: 10.1016/j.ebiom.2015.11.003 (PMC4703710; doi:10.1016/j.ebiom.2015.11.003)
Supplement: Supplementary file 2 — Clinical Protocol [file mmc2.pdf]

## Clinical Protocol:

### 1.1 Preparation and Use of Study Device

In previous studies we isolated multipotent cord blood stem cells (CB-SCs) from human cord blood (Zhao et al., 2006). The CB-SCs display embryonic cell markers (e.g., transcription factors OCT-4 and Nanog, stage-specific embryonic antigen (SSEA)-3, and SSEA-4) and leukocyte common antigen CD45, but they are negative for blood cell lineage markers (Zhao et al., 2006; Zhao and Mazzone, 2010) We identified a hydrophobic material from FDA-approved (USP Class VI) Petri dishes that tightly binds CB-SCs without interfering with their immune modulating capability.

We designed a chamber for co-culture of lymphocytes and CB-SCs that includes nine discs of the material with adherent CB-SCs sandwiched between a top cover plate and a bottom collecting plate. The device was manufactured, assemblied and packaged in a Class 100K clean room. After being sterilized by gamma-irradiation (Cesium-137), the devices are stored at the room temperature in dark cabinets of Class 100K clean room, which is FDA-approved facility for cell isolation and cultures. The materials used to produce the device are FDA-approved for *in vivo* use per the United States Pharmacopeia (i.e., Grade Class VI Plastic). The sterilized device is a single use, which CB-SCs are generated from one cord blood unit for one subject application.

### 1.2 Following steps will be applied for the Preparation and Use of Study Device:

**Step 1: Collection of human cord blood units.**

Human cord blood units derived from healthy donors will be purchased from South Texas Blood & Tissue Center (San Antonio, TX). All cord blood samples are screened for alanine aminotransferase (ALT) levels and for the presence of antibodies to pathogenic antigens (including anti-HCV, anti-HBsAg, anti-HBcAg, anti-HIV-1, anti-HIV-2, anti-Human T-Lymphotropic Virus Types I/II (HTLV I/II), and anti-Syphilis Abs). A sample of blood from the donor's mother must be collected within 30 days prior to donation. After these maternal assessments, only pathogen-free cord blood units are used for isolating CB-SCs. Additionally, at the time of collection, the mother should be in good health and without evidence of active infection that may be transmissible trans-placentally. The medical history should include an assessment of genetic disorders affecting, as a minimum, the genetic mother, father and siblings of the newborn.

Appropriate testing should be performed to demonstrate whether the mother has active Cytomegalovirus (CMV) infection. Cord blood samples will be tested by real time PCR with specific CMV primers to exclude the CMV infection in infants.

**Step 2: Preparation of CB-SC in the device.**

Prior to introducing CB-SCs (Zhao et al., 2006) into the device, cord blood mononuclear cells are isolated from fresh cord blood unit (at least 110 ml/unit) according to the following protocol:

1. Take 50ml tubes and put in holders: normally use 4 tubes;
2. Add HISTOPAQUE®-1077 (Sigma, catalog #10771) to each tube at 20ml/tube;
3. Take an umbilical cord blood unit and sterilize the bag with 70% ethanol;
4. Sterilize the scissors with 70% ethanol and cut the edge of the blood bag;

5. Take 25ml umbilical cord blood and very gently add onto the top of HISTOPAQUE: *the tubes are tilted at 30~45$^o$ angle*;
6. Balance the centrifuge baskets;
7. Centrifuge at 3,000 rpm × 15 min at 4$^o$C temperature, using the Horizontal/Swinging Bucket Rotor (Beckman GPR Centrifuge, GH3.7 Rotor); with a brake at neutral position;
8. Harvest the mononuclear cells at interface, transfer to new 50ml tubes, and then wash with 30-40ml PBS for 1 time, at 2,000 rpm × 10 min at 4$^o$C temperature; discard supernatant;
9. Remove red blood cells by using Lysis buffer (10~15ml/tube) for 15min at room temperature (BD PharmLyse$^{TM}$ Lysis Buffer, BD Biosciences, catalog # 555899);
10. Centrifuge at 1,000 rpm × 5~10 min at 4$^o$C temperature and discard the Lysis buffer; *make sure that most of red blood cells have been lysed and the pellets should be white color*;
11. Wash mononuclear cells with PBS for 2~3 times at 1,000 rpm × 5~10 min at 4$^o$C temperature;
12. Resuspend cell pellets with serum-free cell culture medium (X-vivo, Lonza);
13. Cell counting: adjust cell concentration to $1×10^7$ cells/ml;
14. Plant mononuclear cells in the Device (Tianhe Stem Cell Biotechnologis Inc.) at $1×10^6$cells/ml, 25-30ml/dish in serum-free culture medium;
15. Incubate cells at 37°C, 8% $CO_2$ conditions for 10~20 days.
16. Cell observation: CB-SC are round and attach on the bottom of dishes. If cell density reaches the 80-90% of confluence, CB-SC can be prepared for clinical application.

**Step 3: Testing for endotoxin and mycoplasma.**

1. **Testing for endotoxin.** The supernatant from the culture of CB-SCs will be collected into 1.5 ml sterilized tubes (50 ml/tube). Endotoxin will be tested by using the Limulus Amebocyte Lysate (LAL) assay (Pierce LAL Chromogenic Endotoxin Quantitation Kit, Thermo Scientific). The standard endotoxin level will be < 0.05 EU/ml. This procedure takes about 30 min. Only the Educator apparatus that meets this standard can be used for the clinical trial.
2. **Testing for mycoplasma.** Contamination of mycoplasma in the CB-SC cultures can be tested and excluded by following methods: 1). Regular cell culture: Cord blood-derived mononuclear cells (CBMC) will be simultaneously planted in a small size Petri dish (60 mm) when CBMC are planted in the device of Stem Cell Educator. Cell viability can be monitored under phase-contrast microscope to exclude the contamination of mycoplasma. 2). Real time PCR: We will design specific primers targeting different potential species of mycoplasma contaminations (e.g., M. pneumoniae and M. genitalium). The supernatant from the culture of CB-SCs will be collected into 1.5 ml sterilized tubes (50 ml/tube). The positive control form Charles River (Wilmington, MA) will be run together with the samples. Samples will be run using the Applied Biosystems 7500 FAST to quantification and determination. Only the Educator apparatus that is negative for mycoplasma can be used for the clinical trial.

**Step 4: Preparation of Stem Cell Educator.**

1. Sterilized the hood in a GMP facility for preparation of Stem Cell Educator;
2. Clean out side of device with 70% ethanol gauze and carefully remove all caps;
3. Discard all supernatants inside of device;
4. Add physiological saline to each layer at 20ml/layler, washing each layer and removing all floating cells and debris;
5. Additional wash with physiological saline (20ml/layler);
6. Remove the caps from the top and bottom, replace with Plastisol Horseshoe Y Connectors, and seal with Medical Device Super Glues (Note: these glues meet USP Class 6 criteria for use on medical devices);
7. Add physiological saline to each layer at 15-20ml/layler, and close with caps;
8. Turn around the Stem Cell Educator and check for leaking;
9. Put in the sterilized container;
10. Transport to the clinical site for application.

**Step 5: Treatment with Stem Cell Educator therapy.**

First, all consented subjects will be screened for enrollment in accordance with the Inclusion Criteria and Exclusion Criteria of Stem Cell Educator therapy. Patients will be qualified for enrollment if they meet the 2010 diagnosis standards of the American Diabetes Association and a blood test confirm the presence of at least one autoantibody to pancreatic islet β cells. Preparations for receiving the Stem Cell Educator therapy will include: 1) Subjects should have vegetable diet for 1-2 days prior to the treatment, avoiding the fried foods; 2) Take calcium gluconate (1 tablet, bid), to minimize the risk of hypocalcaemia associated with the administration of heparin during apharesis; 3) In the morning of receiving Stem Cell Educator therapy, subjects can have their breakfast, however with a low amount of drinking water (note: avoid using washroom during the procedure). The detailed procedure of Stem Cell Educator therapy is as follows:

1. **Arrange position**: Set up the blood cell separator, the patient, and Stem Cell Educator appropriately based on the status of patient's vein;
2. **Blood apheresis**: After programming the machine based on the patient's height, body weight, and hematocrit (HCT), a 16-gauge IV needle will be placed in the left (or right) median cubital vein, and the patient’s blood will be passed through a Blood Cell Separator MCS+ (Haemonetics®, Braintree, MA) at 35 mL/min for 6 to 7 hours to isolate lymphocytes in accordance with the manufacturer’s recommended protocol.
3. **Set up connections**: As illustrated in Figure 1, connect the bottle of physiological saline to the Stem Cell Educator, with a connection of filter to adjust the inside pressure of Stem Cell Educator；
4. **Saline pre-wash**: All tubes are primed with saline to optimize the system;
5. **Start treatment**: Lymphocytes isolated from a patient’s peripheral blood are transferred

into the device. CB-SCs begin to treat (or educate) the patient's lymphocytes.

6. **Continuously processing**: In the Stem Cell Educator, lymphocytes separated from a patient's blood are slowly passed through the stacked discs of material with adherent CB-SCs, and lymphocytes can be re-educated by CB-SCs;
7. **Cell return**: Lymphocytes collected through a hole in the bottom plate are returned to the patient, once having enough volume (50 ~ 100 ml). The blood infusion set can be connected to one of the ports of bottom Y connector and prepared for cell return. Initially, the return speed should be low at 40-50 drops / min for 30 minutes; if everything is going well, increase the speed to 70-80 drops / minutes per patient's age and heart function;
8. **Finish apheresis**: In accordance with the program of apheresis, approximately 10,000 mL of blood will be processed during the procedure resulting in approximately two repeated educations for the lymphocyte fraction. All collected lymphocytes, as well as the remaining cells in the bag (rinsed by saline), will be transferred into the Stem Cell Educator for the treatment.
9. **Continue cell return**: Stem cell educator will be rinsed with saline to make sure that most re-educated lymphocytes are returned to the patient.
10. **Finish treatment**: The whole procedure will be finished when the majority of cells are returned to the patient. The remainder of the cells in the infusion set will be kept after sealing with a heat sealer. The bag will be labeled with patient information, date of treatment, and signature. The bag will be stored in -20$^{o}$C or -80$^{o}$C for future reference. Finally, the patient will be monitored for 23 hours in the observation room.

Reference List

Zhao,Y. and Mazzone,T. 2010. Human cord blood stem cells and the journey to a cure for type 1 diabetes. Autoimmun. Rev. 10, 103-107.

Zhao,Y., Wang,H., and Mazzone,T. 2006. Identification of stem cells from human umbilical cord blood with embryonic and hematopoietic characteristics. Exp. Cell Res. 312, 2454-2464.
